# Potential Use of Selected Natural Anti-Microbials to Control *Listeria monocytogenes* in Vacuum Packed Beef Burgers and Their Impact on Quality Attributes

**DOI:** 10.3390/microorganisms13040910

**Published:** 2025-04-16

**Authors:** Angelos Papadochristopoulos, Joseph P. Kerry, Narelle Fegan, Catherine M. Burgess, Geraldine Duffy

**Affiliations:** 1 Teagasc Food Research Centre, Ashtown, D15 DY05 Dublin, Ireland; angelos.papadochristopoulos@teagasc.ie (A.P.); kaye.burgess@teagasc.ie (C.M.B.); 2 Food Packaging Group, School of Food & Nutritional Sciences, University College Cork (UCC), T12 K8AF Cork, Ireland; joe.kerry@ucc.ie; 3 Agriculture and Food, Commonwealth Scientific and Industrial Research Organization (CSIRO), Coopers Plains, Brisbane, QLD 4108, Australia; narelle.fegan@csiro.au

**Keywords:** natural anti-microbials, beef burgers, *Listeria monocytogenes*, beef burger quality, mushroom chitosan, shrimp chitosan, carvacrol, cranberry extract, thyme essential oil, hop extract

## Abstract

This study assessed the potential for natural anti-microbials to control *Listeria monocytogenes* in vacuum packed beef burgers. Minimum inhibitory and bactericidal concentration (MIC and MBC) results for natural anti-microbials (carvacrol; essential oils of thyme, rosemary, clove and cinnamon; hop extract; cranberry extract; cranberry pomace; propolis extract; and chitosan sourced from both shrimp and mushroom) were used to select agents (n = 6) showing the most promise against *L. monocytogenes.* These agents, including chitosan from shrimp and mushroom (a novel source), and cranberry extract, were then tested against *L. monocytogenes* in vacuum packed beef burgers during chilled storage (3 ± 1 °C, 16 days). Following storage (16 d), the number of *L. monocytogenes* in beef burgers treated with chitosan (2.5%), regardless of source, was significantly lower (*p* < 0.05) (1.2 to 1.6 log_10_CFU g^−1^) than in the control samples, while smaller reductions (0.5 log_10_ CFU g^−1^; *p* < 0.05) were noted in samples with cranberry extract (0.625%). While chitosan had no significant impact on HunterLab colour measurements during chilled storage, cranberry extract significantly impacted the colour (*p* < 0.05), resulting in lower L*, a*, and b* values. Observational assessment of colour, odour and the overall quality of the raw meat on opening the pack found that beef burgers with added chitosan (both sources) were acceptable, while those with added cranberry extract received an overall quality score of approximately 5.4, which is above the acceptability threshold (5/10). Overall, the study showed the potential of chitosan to control *L. monocytogenes* in beef burgers, and the advantage of this agent sourced from mushrooms is discussed.

## 1. Introduction

*Listeria monocytogenes* is a psychotropic pathogen, which is considered a ubiquitous microorganism occurring in natural, urban and farm environments [1,2], and has been detected in soil, water, sewage, plants, animals, decaying vegetation, human and animal faeces [3,4,5,6,7]. It can survive and grow at a wide range of temperatures, from −0.4 °C to 45 °C [8], a pH from 4.7 to 9.2 [9] and can survive at high salt concentrations [9]. It thus poses a potential food safety risk as it can survive and potentially grow in chilled foods.

*L. monocytogenes* has been isolated from several fresh and processed food products [9,10], including meat and meat products [8,11,12]. There is potential for cross contamination of meat with *L. monocytogenes* during slaughter and dressing operations, with the hides of bovine animals recognised as a significant source of contamination [11,13,14]. Furthermore, for ready-to-eat meat products, cross contamination can occur during processing, transportation, storage, at retail, or during consumer handling [15]. A study carried out by Khen et al. [11] reported that 29% of raw ground beef products on sale at the retail level in Ireland were contaminated with *L. monocytogenes* at a level of 100–200 colony forming units per gram (CFU g^−1^) (2–2.3 log_10_ CFU g^−1^). Moreover, it is well recognised that the consumption of beef burgers rare or undercooked, a widely reported consumer practice [16,17], increases the risk of illness from products contaminated with any pathogen.

This pathogen can cause invasive listeriosis, especially in infants, elderly people, pregnant and immunocompromised persons, which can lead to meningitis, encephalitis and/or septicaemia, among other infections [8,9]. Several outbreaks of listeriosis related to the consumption of meat products have been reported throughout the years. The biggest outbreak of *L. monocytogenes* was recorded in South Africa in 2017 and was connected to the consumption of a RTE meat product [18,19], whilst a multi-country outbreak in Europe between 2017 and 2019 was also related to the consumption of ready-to-eat (RTE) meat products [20]. All these characteristics make *L. monocytogenes* an important pathogen for the meat industry, and its control is extremely challenging [9].

To ensure the safety of meat products, several preservation methods, such as drying, thermal processing, irradiation, freezing, refrigeration and modified atmosphere packaging or the addition of anti-microbial agents, can be applied [21,22]. However, some of these approaches are not suitable for fresh or processed beef products [23]. Due to the ability of *L. monocytogenes* to grow at chilled temperatures and its facultative anaerobic nature, respectively [9], the addition of an anti-microbial agent is a useful option to support product safety. Equally, consumers are now demanding food products that are considered ‘clean label’ and free of chemical preservatives [24,25,26]. As a result, food industries are adapting meat product formulations to address the expectations of consumers by adding natural anti-microbials in order to ensure safety and extend shelf-life, while also maintaining quality and sensory attributes. Such agents can be added to the product during processing, or they can be incorporated into the packaging material. The origin of natural anti-microbial agents varies and includes essential oils and plant extracts, organic acids, flavonoids, bacteriocins or compounds derived from animals [27]. Some of these anti-microbial agents have already been studied regarding their efficacy against foodborne pathogens and spoilage bacteria in vitro, but also in vivo by being added to meat products or incorporated into packaging films and coatings. For instance, essential oils, including thyme, clove, cinnamon and rosemary, or compounds from essential oils, such as carvacrol, have exhibited anti-microbial activity against *L. monocytogenes* [28,29,30,31,32,33,34]. Moreover, similar activity against *L. monocytogenes* has been observed in studies when plant and fruit extracts such as hop extract, cranberry extract and cranberry pomace have been used [35,36,37,38,39]. Two other agents that have proved to be effective against *L. monocytogenes* are propolis extract and chitosan [40,41,42]. Chitosan, in particular, has shown promising results by exhibiting high anti-microbial activity against other foodborne pathogens in in vitro studies [43,44,45,46,47,48] and when added to meat products [44,48,49,50].

The addition of natural anti-microbials to meat products may, however, have adverse effects on their physical, chemical and organoleptic properties. Colour, texture, lipid oxidation, changes in the pH, production of off-flavours and off-odours and altered sensorial profiles are some parameters that might be impacted [51,52,53,54] and have to be evaluated after the use of natural anti-microbials, whilst the selection of the right concentration is critical [51,52,53,54,55]. Hence, more studies are needed to test the efficacy of those anti-microbial agents against *L. monocytogenes* and how they influence attributes of quality when they are added to meat products, such as burgers stored at low temperature.

This study tested the potential use of several natural anti-microbials in vitro and in vivo against a commonly found foodborne pathogen in meat products. In particular, it focused on compounds, not well investigated for this type of application, including chitosan from mushroom, cranberry extract and hop extract.

The aim of this study was to study the efficacy of several natural anti-microbial agents against *L. monocytogenes,* including their impact on some key quality characteristics in vacuum packaged beef burgers stored at refrigeration temperature (3 ± 1 °C). Initially, a broad group of agents, including essential oils, plant extracts and their compounds (carvacrol; essential oils of thyme, rosemary, clove and cinnamon; and hop extract), agents rich in flavonoids (cranberry extract; cranberry pomace; and propolis extract) and chitosan sourced from both shrimp and mushroom, were screened for activity by determining the minimum inhibitory and bactericidal concentrations of the natural anti-microbial agents against several strains of *L. monocytogenes* under different conditions of growth. Then, the most promising agents were evaluated in beef burgers against a cocktail of strains of *L. monocytogenes* during vacuum packed chilled storage. At the same time, pH, colour parameters, lipid oxidation, visual colour and odour of the beef burgers on opening the pack were also assessed.

## 2. Materials and Methods

### 2.1. Anti-Microbial Agents

Agents examined included carvacrol (≥98%), thyme essential oil, rosemary essential oil, clove essential oil, cinnamon essential oil and chitosan (origin: shrimp, low molecular weight (50–190 KDa), 75–85% deacetylated) (Sigma Aldrich, Arklow, Co Wicklow, Ireland); Beta Bio 40 hop extract (40% b-acids in propylene glycol; brown, clear liquid), (Hopsteiner, Mainburg, Germany); organic propolis extract (Honeygreen S.A.U., Valencia, Spain); mushroom chitosan (Chitoly^®^ AB) from *Agaricus bisporus* (low molecular weight (45 KDa), 98% deacetylated), (Handary S.A. Brussels, Belgium) pure cranberry (*Vaccinium macrocarpon*) extract with 22.5% proanthocyanidin content (BL-DMAC method) and cranberry pomace (Artemis International, Fort Wayne, IN, USA).

### 2.2. Bacterial Strains and Preparation of Inoculum

The anti-microbial activity of all the above agents was tested against five strains of *Listeria monocytogenes* (*L. monocytogenes* NCTC 11994, *L. monocytogenes* EGD-e, *L. monocytogenes* Scott A, *L. monocytogenes* 2081 (mushroom industry isolate, Teagasc culture collection), *L. monocytogenes* 3104 (Teagasc culture collection)) individually. All bacterial cultures were stored on protected storage beads at −80 °C with 80% glycerol. They were resuscitated by streaking a bead onto Tryptone Soya Agar (TSA, Oxoid-ThermoFisher Scientific, Basingstoke, UK). The plates were incubated for 24 h, and then one colony from each strain was transferred in 10 mL Tryptic Soy Broth (TSB, Oxoid-ThermoFisher Scientific, UK) and incubated at 37 °C for 18 h without shaking.

### 2.3. Minimum Inhibitory and Bactericidal Concentrations

The minimum inhibitory concentrations (MICs) and minimum bactericidal concentrations (MBCs) of the anti-microbial agents under different conditions (optimum temperature (T = 37 °C) and neutral pH (pH = 7.3), optimum temperature (T = 37 °C) and low pH (pH = 4.5), low temperature (T = 4 °C) and neutral pH (pH = 7.3), low temperature (T = 4 °C) and low pH (pH = 4.5) were determined using micro-broth dilution assays.

Some of the anti-microbial agents selected required specific preparations before being used for the determination of minimum inhibitory and bactericidal concentrations. Specifically, the Beta Bio 40 hop extract was stored at 4 °C in the dark. A stock solution of 1% *w*/*v* was made with sterile distilled water prior to usage [35]. Propolis extract was dis-solved in 50% ethanol before usage [40], whilst for chitosan, from both sources, stock solutions of 2% *w*/*v* were made by dissolving them in a 1% *v*/*v* glacial acetic acid solution and stirred overnight at room temperature [56,57].

The MIC method used was as described by Rivas et al. [58], whilst some modifications were made for the determination of MIC of propolis extract. In brief, the anti-microbial agents were added to the growth medium (TSB) with 0.05% bacteriological agar (Oxoid-ThermoFisher Scientific, UK). Bacteriological agar was added for stabilizing the solution of TSB with the anti-microbial agents [59]. For the determination of MIC at low pH, the pH of the TSB with 0.05% bacteriological agar was adjusted to pH 4.5 using 0.5 M HCl. Polypropylene microtiter plates with 96 wells (Eppendorf^®^ Microplate 96/U-PP, 96 wells, Sigma Aldrich, Gillingham, UK) were used for the assay. The anti-microbial agents were diluted in two-fold manner across the rows so that each well contained 100 μL of anti-microbial agent in TSB with 0.05% agar.

Cultures of *L. monocytogenes* were diluted to 10^6^–10^7^ CFU mL^−1^ in TSB with 0.05% agar, and 100 μL of culture was added to each well. At least two control wells were included in all assays, consisting of the culture of *L. monocytogenes* in TSB with agar but without any anti-microbial agent added. For propolis extract where 50% ethanol was used, the control also included 10 μL of 50% ethanol. Finally, at least one well was included in each plate as a blank containing TSB with 0.05% agar.

For propolis extract, 90 μL of diluted strains of *L. monocytogenes* and 10 μL of the propolis extract that was dissolved in 50% ethanol were added in the wells of the micro-plate to have a final concentration of 0.02–1% *w*/*v* [40].

The microplates were incubated for 18 h at 37 °C or 4 °C, depending on the condition tested. Measurement of A_595_ of each well was recorded using a microtiter plate reader (Multiskan FC Microplate Photometer, ThermoFisher Scientific, Dublin, Ireland). The MIC was defined as the lowest concentration of the anti-microbial agent in the last well in which growth of *L. monocytogenes* was not detected following incubation [58].

For the determination of the minimum bactericidal concentrations (MBCs), the method outlined by Dygico et al. [60], with some modifications, was followed. By using a 96-pin replicator, 2 μL from each of the wells of the MIC microplates in which no growth was observed were transferred into a new 96-well plate containing 200 µL of Dey/Engley neutralizing broth (Merck Life Science UK Limited (Millipore), Gillingham, UK). The microplates were incubated at 37 °C for 24 h. MBC was defined as the lowest concentration of the anti-microbial agent in the last well where a change in colour of the broth was not observed, representing no growth [60]. All assays were repeated 3 times.

The concentration ranges tested for both MIC and MBC determination were as follows: 0.002–1% *w*/*v* for chitosan from both sources, 0.000195–0.2% *v*/*v* for carvacrol, 0.002–1% *v*/*v* for thyme EO, 0.016–8% *v*/*v* for rosemary EO, 0.004–2% *v*/*v* for clove EO and cinnamon EO, 0.04–20% *w*/*v* for cranberry extract, 0.02–10% *w*/*v* for cranberry pomace, 0.000098–0.4% *v*/*v* for hop extract and 0.02–1% *w*/*v* for propolis extract.

### 2.4. Addition of Natural Anti-Microbials to Beef Burgers (Impact on Survival of L. monocytogenes)

Fresh lean beef meat (approximately 2% fat) (silverside primal cut) and beef fat were obtained from a local beef slaughter plant. The beef meat and beef fat were coarsely minced through the 16 mm plate of a meat grinder (Heavy Duty Buffalo Meat Grinder CD400, Buffalo, UK). Then they were stored at −18 °C and thawed at 5 °C on the pre-trial day. The fat content of the meat was tested by Proximate analysis [61], and the amount of fat to be added to burgers was then calculated in order to yield beef burgers with approximately 20% fat.

The anti-microbial agents with the most promising results in the MIC study were added to beef burgers at different concentrations that were equal or higher to the MIC that was determined. More specifically, chitosan from both sources was added at 0.313%, 0.625%, 1.25% and 2.5%; cranberry extract at 0.625%, 1.25% and 2.5%; carvacrol at 0.1%, 0.2% and 0.4%; thyme EO at 0.125%, 0.25% and 0.5%; and hop extract at 0.1%, 0.2% and 0.4%.

Cultures of the five strains of *L. monocytogenes* were incubated at 37 °C for 18 h without shaking. Subsequently, the cultures were centrifuged at 5000 rpm for 10 min at 4 °C. The supernatant was discarded, and the pellet was washed and resuspended with 10 mL Phosphate-Buffered Saline (PBS) (Oxoid-ThermoFisher Scientific, UK). All strains were combined into a single tube, and then the cocktail of *L. monocytogenes* was added to minced meat to yield a concentration of approximately 3.8 log_10_ CFU g^−1^.

The ground beef meat, the fat, the anti-microbial agent and the cocktail of *L. monocytogenes* were manually mixed in a sterile aluminium tray and then passed through the 5 mm plate of the grinder. The control samples were prepared using the same procedure, but without the addition of the anti-microbial agent. Inoculated ground meat (160 g) was shaped into burgers (10 cm diameter, 2 cm thickness) using a burger press (Buffalo manual hamburger machine, Buffalo, UK). The burgers were placed in PA-PE-EVOH bags (O_2_ transmission: 2 cm^3^/m^2^/24 h) (Versatile Packaging Ltd., Co Monaghan, Ireland), vacuum packaged (99% vacuum) (Eco Pack, Ecovac, Italy) and stored at 3 ± 1 °C for 16 days.

Microbiological analysis of the burgers was conducted on days 0, 4, 8, 12 and 16 of storage. Each day, two burgers were analysed, and 25 g from each burger was transferred aseptically in a stomacher bag (Separator 400 Blender Bag, Grade, UK) and homogenized with 225 mL Maximum Recovery Diluent (MRD) (Oxoid-ThermoFisher Scientific, UK) for 60 sec in a lab blender (BagMixer 400 P, Interscience, France) at room temperature. The population of *L. monocytogenes* was determined by serial dilutions in MRD and plating onto Listeria Selective Agar (Oxford Formulation) (Oxoid-ThermoFisher Scientific, UK) followed by incubation at 37 °C for 24–48 h. An overlay method was also used to facilitate the recovery of any stressed and injured cells that might be present. The samples were serially diluted in MRD and then plated onto TSA. The TSA plates were incubated at 30 °C for 2 h to allow injured cells of *L. monocytogenes* to resuscitate, and then a layer of selective agar (Oxford Formulation) was poured on top of the recovered cells. The plates were then further incubated at 37 °C for 24–48 h. The injury ratio was calculated by using the following equation [62]:

Injury ratio (%) = [1 − (N_OXFORD_/N_TSA_)] × 100

where N_TSA_ (CFU g^−1^) was the counts in the nonselective medium overlaid with the selective agar; N_OXFORD_ (CFU g^−1^) was the counts in the selective medium.

The experiments were conducted twice (n = 2 × 2), and the data were analysed as described below.

### 2.5. Addition of Natural Anti-Microbials to Beef Burgers (Physicochemical Analysis and Observational Study on Visual Colour and Odour)

The anti-microbial agents that exhibited the best results against *L. monocytogenes* in beef burgers were added at the highest concentrations tested to the burgers. Then, the burgers with the natural anti-microbials, as well as the control samples, were placed in PA-PE-EVOH bags, and they were vacuum packaged (99% vacuum) and stored at 3 ± 1 °C for 16 days. For all analyses below, two uninoculated burgers were tested on days 0, 8 and 16 of chilled storage.

The pH of the burgers was measured with a pH meter (FiveGo pH meter F2, Mettler Toledo, Switzerland), with a glass electrode that was placed directly into the burger. The pH meter was first calibrated using buffer solutions at pH 7 and pH 4. The pH of each burger was measured at two different locations within them.

The colour parameters of the burgers were assessed through the packaging using a HunterLab UltraScan Pro (Hunter Associates Laboratory, Inc., Reston, VA, USA) dual beam xenon flash spectrometer, with a viewing port of 25.54 mm and illuminant D65, 10°. L* (lightness/darkness), a* (redness/greenness) and b* (yellowness/blueness) were measured at three different locations of the burger. The colourimeter was calibrated before use with a white tile covered with the same packaging film as the burgers.

Lipid oxidation in beef burgers was determined using the Thiobarbituric acid reactive substances (TBARSs) method, as described by Moran et al. [63], with some modifications. Briefly, approximately 3 g of burger on each day of sampling was weighed in duplicate and homogenized with 20 mL MilliQ water using an IKA T25 digital Ultra-Turrax homogenizer (IKA, Staufen, Germany) at 13,500 rpm for 30 s. Then, 5 mL of 25% trichloroacetic acid (TCA) solution was added, the tubes were mixed and they were left at 4 °C for 15 min. Afterwards, they were centrifuged at 3500 rpm for 30 min. The following steps were conducted according to the protocol of Moran et al. [63]. Regarding the samples with cranberry extract, a blank with 3.5 mL from the filtrate and 1.5 mL MilliQ water was used in order to remove any interference in the absorbance caused by the natural red colour of the sample. The absorbance was measured in duplicate for each sample on a UV–Vis Spectrophotometer (Jenway 6300, Cole-Palmer Ltd., St Neots, UK) at 532 nm. Standard malondialdehyde (MDA) working solutions were made to create a standard curve based on the method described by Maraschiello et al. [64]. The results were expressed in mg of malondialdehyde (MDA) per kg of burger.

Observational studies on visual colour, off-odour, overall odour and overall quality of the raw beef burgers were conducted in the laboratory by a team of four trained panellists. The panellists were trained by the Sensory Scientists at Teagasc, Ashtown, and carried out the observational study on the raw meat based on the protocols and scoring sheets of the American Meat Science Association Standards for sensory analysis [65]. The colour was assessed before opening the package using the scale below: 1 = bright purple-red; 2 = dull purple-red; 3 = slightly brownish-red; 4 = moderately brownish-red; 5 = brown; 6 = dark purple. After opening the packaging of the burger, the odour attributes (off-odour and overall odour) were assessed on a scale of 1 (none) to 8 (very strong). The overall quality of beef burgers was assessed using a 10-point quality scale from 1 (reject) to 10 (match), with scores ≤ 5 to be considered as unacceptable [66]. The same procedure for visual colour, odour, and overall quality was repeated 30 min after opening the package [67].The experiments were repeated three times with two technical replications (n = 3 × 2).

### 2.6. Statistical Analysis

The impact of chitosan from both sources and cranberry extract on the survival of *Listeria monocytogenes* in beef burgers was analysed using a three-way analysis of variance (ANOVA), while a two-way ANOVA was applied to the data from carvacrol, thyme EO and hop extract. In both cases, comparison of means was performed using Tukey’s HSD test. The data on the impact of natural anti-microbials on pH, colour, lipid oxidation (TBARS), visual colour, off-odour, overall odour and overall quality were analysed using a two-way ANOVA, and Tukey’s HSD test was used for post hoc comparisons. All tests were performed with RStudio (Version 2023.12.1+402, Posit Software, Boston, MA, USA), and statistical significance was defined as *p* ≤ 0.05. The detection limit for the determination of *L. monocytogenes* in beef burgers was 1 log_10_ CFU g^−1^. When samples had counts lower than the detection limit, an arbitrary value equal to half of the detection limit was used for the final calculations of bacterial populations [68].

## 3. Results

### 3.1. Minimum Inhibitory and Bactericidal Concentrations of Natural Anti-Microbials Against L. monocytogenes

The MIC and MBC for all anti-microbial agents tested against the five strains of *L. monocytogenes* are presented in Table S1 in the Supplementary Materials. The pH (4.5, 7.3) and temperature (4 °C, 37 °C) had a significant impact on results. No growth was observed after 18 h at 4 ± 1 °C or when the bacteria were added in the medium with a low pH (4.5). As a result, the determination of the MIC under these conditions was not possible. Moreover, in some cases, the MBC was out of the range of the concentrations studied, and determination was not possible.

The activity of all tested agents against *L. monocytogenes* varied depending on the concentration used and environmental conditions, with some inter-strain differences and variability reported. Under optimum conditions of 37 °C and pH 7.3, chitosan, whether from shrimp or mushroom, had a similar MIC (0.016–0.031%), while MBC varied from 0.031% to 0.125% depending on the strain. Under the same conditions of growth, cranberry extract had an MIC of 0.625% with no variability between the strains and MBC at the level of 1.25–5%. Meanwhile, the MIC of carvacrol ranged from 0.05% to 0.1%, and the MBC was steady at 0.1%. For hop extract, the MIC under optimal growing conditions was the lowest among the agents tested (0.001–0.003%), whilst the MBC varied from 0.2% to concentrations higher than 0.4%.

### 3.2. Impact of Natural Anti-Microbials Added to Beef Burgers on the Survival of L. monocytogenes

Following the initial MIC and MBC study, the activity of selected anti-microbial agents (n = 6) against *L. monocytogenes* inoculated into beef burgers was assessed. The selection criteria for the agents was based on the MIC of the compounds, their potential impact on the organoleptic properties of the beef burgers and the innovative characteristics of the agents tested. The agents chosen were carvacrol, thyme EO, hop extract, cranberry extract and chitosan from both sources. The concentration selected for carvacrol, thyme EO and cranberry extract was based on the MIC. Three concentrations were tested, the minimum inhibitory concentration and two higher concentrations, two times the MIC value and four times the MIC. The concentrations of hop extract selected were much higher than the MIC because initial studies at concentration ranges of 1× MIC–4× MIC showed no anti-microbial effect against *L. monocytogenes*. Thus, concentrations of 0.1%, 0.2% and 0.4% were selected, as having been shown effective in other studies [36]. Regarding chitosan from both sources, the concentrations that were chosen were also higher than the MIC at 0.313%, 0.625%, 1.25% and 2.5%. Chitosan from crustaceans has previously been used at concentrations of 0.4–1% in meat products to control *L. monocytogenes* [41,42,50]. However, it was not known whether chitosan from mushrooms would have a similar impact at that concentration range (0.4–1%). Additionally, the recommended dosage of chitosan from mushroom was up to 2%, but the exact concentration and efficacy depend on the specific product, and its effectiveness against *L. monocytogenes* in beef burgers at this concentration was unknown. Therefore, a wider range of concentrations (0.313–2.5%) were applied, and it was decided that the same concentrations should also be used for chitosan from shrimp.

Furthermore, both types of chitosan were dissolved into 1% acetic acid for the MIC and MBC study, while they were incorporated as a dry powder into the meat.

The impact of natural anti-microbials added to beef burgers on the survival of *L. monocytogenes* is presented in Table 1 and Table 2 below.

At the highest concentration used, chitosan from both sources, cranberry extract and carvacrol, showed increasing anti-microbial activity in beef burgers over storage, reaching their highest effectiveness by day 12 at 3 °C. Particularly, chitosan from both sources exhibited the highest bactericidal activity against *L. monocytogenes* in beef burgers compared to the other anti-microbial agents studied when the concentration was increased to 2.5%. After 16 days of storage, the number of *L. monocytogenes* in beef burgers treated with chitosan (2.5%), regardless of source, was significantly lower (*p* < 0.05) (1.2 to 1.6 log_10_ CFU g^−1^, overlay method) than in the control samples. The injury level of cells was also high, with approximately 60% injury being noted after 16 days of chilled storage. Additionally, on day 16 of refrigerated storage, chitosan from mushroom demonstrated significantly higher anti-microbial activity (*p* < 0.05) at all tested concentrations compared to chitosan from shrimp.

After 16 days of storage, smaller reductions (*p* < 0.05) of approximately 0.5 log_10_ CFU g^−1^ and 0.6 log_10_ CFU g^−1^ (overlay method) were reported in samples with cranberry extract (0.625%) and carvacrol (0.4%), respectively, compared to the control. The highest levels of injury noted were 36% in beef burgers with cranberry extract (1.25%) at the end of storage, while carvacrol (0.4%) resulted in 46% injured cells on day 0. Minimal impact on *L. monocytogenes* was noted after the addition of thyme EO to the beef burgers, with 0.5% thyme EO resulting in a lower population (*p* > 0.05) by 0.27 log_10_ CFU g^−1^ (overlay method) in comparison to the control samples at the end of storage period. An injured cell level of 23% was noted at mid-storage in burgers with 0.25% thyme EO. Finally, hop extract did not show any anti-microbial effect against *L. monocytogenes* at any concentration tested. The level of injured cells recovered highlights the importance of using the overlay method, which facilitated their recovery.

### 3.3. Impact of Natural Anti-Microbials on the Quality Attributes of Beef Burgers

The effect on quality parameters of the best performing agents from the initial study, chitosan from either source, cranberry extract and carvacrol, which exhibited the highest anti-listerial activity in beef burgers, was also studied.

Table 3 shows that the pH of the beef burger was significantly impacted by the addition of the anti-microbial agent. The addition of chitosan (both sources) resulted in a significantly higher pH (*p* < 0.05) on days 0, 8 and 16 compared to the control. Beef burgers with the added cranberry extract (2.5%) had a significantly lower pH (*p* < 0.05) on days 0, 8 and 16 compared to the control, whereas carvacrol had no impact on pH.

The impact of natural anti-microbials on the colour parameters (L*, a*, b*) of beef burgers as measured by the HunterLab colourimeter is presented in Table 4. The most significant impact on colour was noted in samples with added cranberry extract, with a significantly lower (*p* < 0.05) L* and b* values observed at all times points (0, 8 and 16 d) in comparison with the control and other tested agents. Additionally, a* values were significantly lower (*p* < 0.05) at the end of chilled storage. Chitosan, irrespective of source, had no significant impact (*p* > 0.05) on the L* values of burgers, while the a* and b* values were significantly lower (*p* < 0.05) than the control only on day 0 of chilled storage. The addition of carvacrol to the burgers had minimal impact on their colour parameters, with L* values being significantly higher (*p* < 0.05) than those of the untreated burgers on days 8 and 16 of storage.

The level of lipid oxidation (TBARS) was low (<1 mg MDA kg^−1^) for all samples irrespective of treatment throughout the chilled storage.

The results of the observed visual colour and odour assessment of the beef burgers on opening the pack and 30 min later are presented in Table 5 and Table 6, with the effect sizes (η^2^) listed in Tables S2 and S3 in the Supplementary Materials. As observed in the colour analysis with the Hunterlab colourimeter, the panel also scored the samples with cranberry extract as significantly different (*p* < 0.05) from the control and other agents, having a dark purple colour at all time points examined. The results were similar on opening and 30 min later. The strong and significant impact of anti-microbial agents on visual colour, as shown in Tables S2 and S3, was only due to the samples with cranberry extract. Excluding these samples results in a non-significant effect (*p* > 0.05) of the agents on the visual colour of the burgers.

After 16 days storage, in general, no off-odours were observed, except for the samples with chitosan from shrimp, where there was a trace of off-odour reported. In terms of the overall odour of the burgers, substantial variation was observed between the panellists. The addition of 0.4% carvacrol to burgers led to a moderate–definite odour. None of the other anti-microbials tested produced an odour that differed significantly compared to control samples. The overall quality scores of the burgers were strongly influenced by the natural anti-microbial agent used (η^2^ = 0.35, *p* < 0.0001). The ratings for the control and burgers with chitosan from either source were acceptable during the chilled storage, while beef burgers with added cranberry extract and carvacrol scored above 5 on the 10-point scale but had significantly lower scores (*p* < 0.05) compared to the control throughout the storage period.

## 4. Discussion

This study examined the anti-microbial activity of a broad range of natural anti-microbial compounds at various concentrations against *L. monocytogenes* in beef burgers, as well as their impact on measured quality attributes. As expected, there was high variability in results between the different agents and the concentration used.

### 4.1. Minimum Inhibitory and Bactericidal Concentrations of Natural Anti-Microbials Against L. monocytogenes

Initially, the anti-listerial activity of several agents (n = 11), including chitosan from two sources (shrimp and mushroom), a number of essential oils and carvacrol, were examined. The MIC of chitosan from both sources was from 0.016% to 0.031%. Similar findings have been reported in the studies of Lamas et al. [69] and He et al. [46], with the MIC at the range of 0.013% to 0.025% and 0.023%, respectively. When the pH is higher than the pKa of chitosan (6.3–6.5), its anti-microbial mode of action is based on its chelating properties, and it involves destabilization and disruption of the cell membrane [70,71]. At the molecular level, the downregulation of genes involved in RNA and protein synthesis and the metabolism of carbohydrates, amino acids, nucleotides and nucleic acids, lipids and coenzymes has also been reported [72].

Cranberry extract also showed good efficacy against *L. monocytogenes*. The MIC of the American cranberry extract was 0.625%, a value that is similar to the MIC (0.313% to 0.625%) of swamp cranberry extract reported in the study of Stobnicka and Gniewosz [38]. Although these are two different varieties of cranberries, both contain high amounts of polyphenols, such anthocyanidins and proanthocyanidins [73,74,75]. The antibacterial activity of polyphenols involves several mechanisms, such as biofilm inhibition, inhibition of cell wall biosynthesis, inactivation of lipopolysaccharides (LPSs), destruction of lipid bilayers, inhibition of cell membrane functions, pore formation and leakage of intracellular material, inhibition of enzyme activity, inhibitions of DNA gyrase and inhibition of DNA and RNA synthesis [76]. Additionally, cranberry extract can disrupt the cell wall and cell membrane, leading to leakage of cytoplasmic content in Gram-negative and Gram-positive bacteria [77]. At the transcriptional level, upregulation of genes related to cell wall biosynthesis and cell wall stress has also been reported [78].

Carvacrol, an anti-microbial compound found in oregano and thyme essential oils, among other oils and plant extracts [79], had an MIC against the five strains of *L. monocytogenes* between 0.05% and 0.1%. This was in accordance with the MIC (0.02% to 0.1%) reported by Rivas et al. [58], Veldhuizen et al. [30] and Arioli et al. [80]. For thyme EO, the MIC (0.125%) was lower than the concentration reported in other studies. According to Mazzarrino et al. [81], the MIC of thyme EO fluctuated among the strains between 0.25% and 0.5%, whilst in the studies of Pesavento et al. [82] and Gouveia et al. [34], the MICs against *L. monocytogenes* were 0.25% and 0.39%, respectively. Both anti-microbial agents can destabilize and disrupt the cell membrane, leading to leakage of cellular material and eventually cell death [83,84,85].

Hop extract exhibited a low MIC against *L. monocytogenes* (0.002%), which was similar to the values determined by Kramer et al. (0.00125% and 0.00032%) [35,36]. The anti-microbial mechanism of the hop extract β-acids used in this study involves the inhibition of the active transport of sugars and amino acids, leading to disruption of cellular respiration, replication, transcription and translation [86].

### 4.2. Impact of Natural Anti-Microbials Added to Beef Burgers on the Survival of L. monocytogenes

The addition of chitosan from either source to beef burgers, especially at concentration levels of 1.25% and 2.5%, led to a strong bactericidal effect against *L. monocytogenes*, with significant reductions (>1 log_10_ CFU g^−1^) compared to the control, observed at the end of chilled storage (16 d at 3 °C).

There were some differences noted in the activity of chitosan from mushroom and shrimp, especially at higher concentrations, with chitosan from mushroom exhibiting significantly higher (*p* < 0.05) anti-listerial activity at the end of chilled storage. These differences may be attributed to the fact that chitosan from mushroom had lower molecular weight and higher degree of deacetylation than chitosan from shrimp, which could contribute to its higher efficacy against microorganisms [56,87]. Additionally, Chien et al. [88] observed slightly higher anti-microbial activity of chitosan from mushroom compared to chitosan from shellfish (crab).

Findings in the literature that are consistent with the anti-listerial activity of chitosan observed in the current study were reported by Economou et al. [41] and Antoniadou et al. [42], who dipped beef pieces and ready-to-eat bovine meatballs, respectively, in 1% chitosan solution (crab). Economou et al. [41] reported approximately 1.2 log_10_ CFU g^−1^ lower *L. monocytogenes* counts for samples stored under vacuum compared to the control at the end of chilled storage, whilst Antoniadou et al. [42] noted a 2.16 log_10_ CFU g^−1^ difference with the untreated samples after 21 days of storage at 4 °C.

There have been limited studies on the anti-microbial efficacy of chitosan when it is added directly into meat products without dissolving it into an acidic solution first. Chitosan can be completely dissolved in aqueous solutions with a pH lower than 6 [89]. In this study, it was applied directly in the burgers without being dissolved in 1% glacial acetic acid, whilst for the experimental work of MIC and MBC it was dissolved in the acetic acid solution because the pH of TSB was approximately 7.3. Incili et al. [50] also assessed the anti-microbial effect that the direct addition of 0.4% chitosan (shrimp) would have in beef meatballs with 15% fat. *L. monocytogenes* counts were significantly (*p* < 0.05) lower by 1.8 log_10_ CFU g^−1^ in contrast to untreated meatballs after 10 days at 4 °C. Storage atmosphere may have an impact on efficacy, and Economou et al. [41] noted higher anti-listerial activity in the meatball samples stored in aerobic conditions.

The majority of studies to date on chitosan are on crustacean-sourced agents, and this is the first study to show that the anti-microbial activity of chitosan from mushrooms is equivalent or performed better when added to beef burgers. This is a significant finding as chitosan from mushrooms would not be considered to be an allergenic compound in comparison to chitosan sourced from crustaceans [90]. Additionally, with growing concerns about the environment, valorisation of by-products from mushroom production to extract chitosan would contribute to a circular bioeconomy [91]. While this study was limited to evaluating the impacts on quality attributes, and indeed showed no significant effect, the next step would be to validate that the eating quality of beef burgers is not negatively impacted by chitosan from mushrooms, although there is no indication in the literature suggesting that this would occur [53,92].

Cranberry extract showed significant (*p* < 0.05) anti-listerial activity at all concentrations tested at the end of storage period. High efficacy against *L. monocytogenes* has also been reported in other studies. Qiu and Wu [39] observed a 2.3 log_10_ CFU g^−1^ difference in the population of the pathogen compared to the control at the end of storage at 7 °C when a high concentration of cranberry concentrate (10%) was added to beef burgers, whilst Stobnicka and Gniewosz [38] noted a 3 log_10_ CFU g^−1^ reduction after 2 days of chilled storage in pork burgers containing 2.5% swamp cranberry. The difference in the efficacy of cranberry extract between our study and the study of Stobnicka and Gniewosz [38] could be attributed in the concentration of bioactive compounds that contribute to the anti-microbial activity of cranberry.

The anti-listerial activity of carvacrol and thyme essential oil in beef burgers was minimal for most of the concentrations used. The activity of carvacrol was dose-dependent, with a significantly lower population (*p* < 0.05) of *L. monocytogenes* reported at the end of the storage period when 0.4% carvacrol was used. At the same time, lower concentrations of 0.1–0.2% (1× MIC–2× MIC) exhibited a minimal anti-listerial effect in beef burgers. Veldhuizen et al. [30] reported similar results, with no effect being observed when carvacrol was added to beef steak tartare stored at 10 °C. They also reported lower bactericidal activity of carvacrol at low temperatures (10 °C). Additionally, *L. monocytogenes* was completely eliminated in beef marinated in teriyaki sauce containing 0.5% carvacrol after 7 days of refrigerated storage [93]. These studies would support the need for the use of higher concentrations (0.4%) to observe any anti-listerial activity in beef burgers. Generally, higher concentrations of essential oils and their compounds than the MIC determined are needed when applied in vivo (i.e., food products) in order to exhibit the same anti-microbial activity [84]. Hence, the higher concentrations needed in the present study could also be attributed to the interaction with food components, matrix, and fat and protein content [30,84].

Thyme EO had minimal impact on the survival of *L. monocytogenes* in the beef burgers. A statistically significant (*p* < 0.05) reduction compared to the control was observed only after 16 days of storage at 3 °C when added at 0.5%, as determined with the method of direct plating, which does not detect injured cells. Pesavento et al. [82] also reported bacteriostatic activity of thyme EO when it was added at a concentration of 0.5% in beef meatballs stored at low temperature. In the same study, a strong bactericidal effect against *L. monocytogenes* was achieved when higher concentrations (1% and 2%) were applied. On the other hand, when Solomakos et al. [28] added 0.6% thyme EO in minced beef meat, an approximately 3 log_10_ CFU g^−1^ difference of *L. monocytogenes* compared to the control after 12 days at 4 °C was observed. The differences in the findings of the studies could be attributed to the use of sterile meat from Solomakos et al. [28]. The initial meat microflora might assist *L. monocytogenes* to survive during chilled storage in the presence of 0.5% thyme EO. Furthermore, the absence of strong anti-listerial activity of thyme EO in the present study might be attributed to its composition. The EO tested were supplied by a commercial manufacturer (Sigma Aldrich) and met the company’s specifications; hence, further chemical analysis of the thyme essential oil was not conducted. It is possible that some variation in its composition could have potentially influenced its anti-microbial activity.

Despite the fact that the MIC of hop extract against *L. monocytogenes* was very low, much higher concentrations of hop extract were added to the burgers. Additionally, while anti-listerial activity was observed in the in vitro studies, it was not detected in the in vivo experiments. The findings of the present study did not align with those of Kramer et al. [35,36], who reported that *L. monocytogenes* growth in pork with marinade containing 0.5% hop extract was inhibited [35], and that the application of low concentrations (0.04% and 0.08%) of hop extract in bologna sausages exhibited significant anti-microbial activity against *L. monocytogenes* [36]. The quantity of fat in the burgers (20%) may explain the findings of our study; although, Kramer et al. [36] proved that hop extract is effective in the presence of high fat levels (20% and 26%) but at the same time exhibits its highest efficacy in low-fat products [35]. Hop β-acids, which are hydrophobic [94], were probably dissolved in the fatty phase of the burger, and, as a result, they were not in contact with the pathogens that were in the aqueous phase [35]. Furthermore, the type of meat product could also play a major role in the efficacy of hop extract. Comminuted meat products might not be the ideal product for hop extract to exhibit its anti-microbial activity. Finally, hop extract was dissolved in water prior to use; thus, the water activity of burgers was increased with the addition of the extract, and, as it is noted in Kramer et al. [36], low water activity favours its anti-listerial activity. In that study by Kramer et al. [36], an equal quantity of ice was removed from the recipe of bologna sausages when hop extract was used.

The effectiveness of some natural agents, especially chitosan from both sources, is comparable to the anti-microbial activity of synthetic preservatives in meat products. For example, the addition of sodium nitrate and sodium sulphite to minced meat at the maximum concentrations allowed by legislation resulted in approximately 1.2 log_10_ CFU g^−1^ lower counts of *L. monocytogenes* than control samples on day 9 of cold storage [69], which is lower than the reduction observed with 2.5% chitosan from both sources in the current study. Additionally, sodium lactate (1.8 and 2.5%) and sodium diacetate (0.1%) reduced the growth rate of *L. monocytogenes* in a meat emulsion stored at 5 °C for 30 days [95]. According to Lim and Mustapha [96], 0.5% cetylpyridinium chloride, 0.12% acidified sodium chlorite and their combination led to similar or higher differences in the population of the pathogen between treated and control samples in fresh beef on day 14 of storage at 4 °C, compared to those observed in the present study. In contrast, 0.1% potassium sorbate exhibited limited effectiveness.

### 4.3. Impact of Natural Anti-Microbials on the Quality Attributes of Beef Burgers

The addition of some of the natural anti-microbial agents influenced the meat pH throughout storage. pH is a crucial factor for the quality of meat, as it can impact several attributes, such as microbial spoilage, lipid oxidation, colour and flavour [97,98,99,100,101,102]. The pH of the untreated burgers was approximately 5.7. However, the addition of chitosan from either source increased the pH sharply (*p* < 0.05) to approximately 6.6 from day 0. Suman et al. [103], Amoli et al. [104] and Hautrive et al. [105] made similar observations with higher pH values for ground beef and low-fat beef burgers, respectively, in comparison to the control. More specifically, in the studies of Amoli et al. [104] and Hautrive et al. [105], it was noted that the high pH value of the chitosan contributed to the shift in the pH. Chitosan is produced by deacetylation of chitin using a strong alkali treatment (45% NaOH) that influences its properties [90,106]. These findings are in contrast with the results of the study of Chounou and colleagues [53], in which beef burgers with 1% chitosan directly added to the meat and stored aerobically or with O_2_ scavenger in the packaging at 4 °C had a similar pH to the control (5.8 to 6.0). Additionally, lower pH values in the course of storage compared to the control were reported for pork patties coated with chitosan film and beef dipped in 0.5% chitosan solution and stored aerobically at 4 °C [107,108].

Cranberry extract also affected the pH of the burgers. Cranberry has a naturally low pH value, which is around 2.6 for the juice [109]. As a result, the pH of burgers with 2.5% cranberry extract was around 5.31 on day 0, reaching 5.22 at the end of chilled storage. Higher acidity and lower pH in contrast to the control was noted in the study of Stobnicka and Gniewosz [38] in which swamp cranberry extract was added to pork burgers. Additionally, according to Wu et al. [110], 2.5% cranberry concentrate with a pH of 2.2 reduced the pH of ground beef from 5.6 to 4.9.

The addition of carvacrol to beef burgers had a minimal impact on pH values. Moon et al. [93] and Hulankova et al. [111] reported the same trend for beef marinated in sauce with different levels of carvacrol and minced beef with 0.2% oregano essential oil (EO) containing 72% carvacrol, respectively, during chilled storage.

The impact of chitosan from both sources on the colour attributes of beef burgers was not significant. Mixed results regarding the effect of chitosan on the colour of meat have previously been reported in the literature. Higher values of lightness (L*) and yellowness (b*) were observed throughout the storage of beef dipped in 0.5% chitosan solution, low-fat burgers with chitosan at different concentrations (0.5–2%) and pork burgers with 0.25–1% chitosan by Khan et al. [108], Amoli et al. [104] and Sayas-Barberá et al. [112], respectively. The higher lightness that was observed mainly in the samples with chitosan from shrimp, especially on days 8 and 16, could be attributed to the water binding properties of chitosan as it traps the water inside the burger [104,113]. Generally, higher water content in meat products leads to higher reflection and higher L* values [104,114]. Additionally, the whitish-beige colour of chitosan might affect the b* values, leading to the higher yellowness in burgers [104,112]. A similar observation was made in the current study, but a slight increase (*p* > 0.05) was reported. In contrast, Chounou et al. [53] noted that the lightness (L*) decreased throughout the storage for the ground meat treated with chitosan (1%) in contrast to the control samples. It is noted that the method of colour evaluation differed significantly between the two studies. Chounou et al. [53] measured the colour parameters 30 min after the package with the ground meat was opened, whilst in the current study the assessment was performed through the packaging directly after the burgers were removed from chilled storage. According to the studies of Sayas-Barberá et al. [112], Amoli et al. [104] and Darmadji and Izumimoto [115], the addition of chitosan improved the red colour (a*) in pork burgers, low-fat beef burgers and minced beef, respectively. On the contrary, Khan et al. [108] observed significantly lower values of redness (*p* < 0.05) in beef coated with chitosan. In our study, an increase of redness for the samples treated with chitosan from both sources and decrease for the control in the course of storage was observed; however, at the end of storage, the a* value was still higher for the control compared to the other samples. The use of vacuum packaging in our study might have influenced the impact of chitosan on redness, as reported in the study of Suman et al. [103], where no significantly higher a* values (*p >* 0.05) were observed between beef patties with 1% chitosan and control samples under vacuum after 5 days at 1 °C.

Cranberry extract caused significant changes in the colour of burgers. Lightness, redness and yellowness were significantly lower (*p* < 0.05) in the course of chilled storage compared to the control due to the dark-purple colour of the burgers. The impact of cranberry on the colour properties of ground meat or prime cuts has not been studied extensively in the literature, presumably because its effect is clear. When it was added in other types of processed meat products, such as frankfurters, it led to a darker colour internally and externally [52].

In the majority of cases, the colour of burgers was not heavily affected by the addition of 0.4% carvacrol. Other studies support this finding, with Moon et al. [93] reporting no significant impact (*p* > 0.05) on L*, a* and b* values of meat over the storage period by the addition of 0.3% and 0.5% carvacrol to beef marinade. Nevertheless, according to Hulankova et al. [111], oregano EO negatively influenced the colour, with substantially higher (*p* < 0.05) lightness, redness and yellowness throughout refrigerated storage.

Lipid oxidation in meat products is an indicator of spoilage, with the development of off-odours, off-flavours and changes in colour and texture [116]. According to the literature, the minimum level of detection of rancidity in meat products that leads to rejection of the product varies, and values of 0.5 mg MDA kg^−1^ meat, 1 mg MDA kg^−1^ meat and 2 mg MDA kg^−1^ meat have been reported [117,118,119]. Moreover, the threshold seems to be higher, at the level of 2 mg MDA kg^−1^, in beef than in pork products [119]. All burgers studied were below that limit for the 16 days of storage at 3 °C.

Chitosan exhibits anti-oxidant properties [120,121] apart from anti-microbial ones [122]; thus, it was expected that lipid oxidation in the samples treated with 2.5% chitosan from either source would be remarkably lower compared to the control in the course of chilled storage. Nevertheless, the positive impact of chitosan from either source on lipid oxidation in burgers was not evident. TBARS values were lower than the control, but not significantly (*p* > 0.05). The lipid oxidation in untreated beef burgers stored under vacuum was not at a high level, and it was always below the rejection threshold during the chilled storage. As a result, the addition of chitosan did not make a big difference. This was observed in the study of Duran et al. [123], in which beef coated with 2% chitosan solution was vacuum packed and stored at 4 °C for 45 days. Only at day 15 of chilled storage was there a significant difference (*p* < 0.05) in lipid oxidation between treated and control samples. In contrast, Suman et al. [103] reported significantly lower (*p* < 0.05) TBARS values for ground beef with 1% chitosan in contrast to the control at day 0 and day 3 of storage at 1 °C under vacuum. The vacuum packaging retards the oxidation of lipids because it removes the oxygen from the packaging, which is needed for the chemical reaction [124]. According to findings from other research groups [53,54,104,107,108,115], the use of chitosan in the form of a coating or film or by direct addition of it to meat products was an effective solution to reduce lipid oxidation when they were stored in aerobic conditions. On the other hand, Hautrive et al. [105] did not observe a significant difference (*p* > 0.05) in lipid oxidation between burgers with 4% chitosan and control samples during frozen storage.

Cranberry’s anti-oxidant properties [75] make it an ideal agent to inhibit lipid oxidation in processed meat products. Lower levels of oxidation for burgers with 2.5% cranberry during storage at 3 °C were reported. Similar results were reported from Lee et al. [125]. In that study, various phenolic compounds extracted from cranberry concentrate juice powder inhibited lipid oxidation in cooked ground pork (30% fat) by 22% to 81% throughout cold storage.

Carvacrol proved to be an effective method to inhibit lipid oxidation in beef burgers by decreasing significantly (*p* < 0.05) the level of lipid oxidation at day 0, whilst maintaining that low level during storage. Carvacrol and essential oils and their components generally possess anti-oxidant properties [126]. Bellés et al. [127] noted reduced oxidation levels in lamb burgers with 0.03% and 0.1% carvacrol in comparison to the control throughout storage. These findings were not confirmed by Moon et al. [93], who did not observe a significant impact (*p* > 0.05) on limiting lipid oxidation in marinated beef during refrigerated storage.

The Instrumental analysis of quality attributes was supported by an observational visual colour and odour assessment of the raw burgers by four panellists. No significant differences (*p* > 0.05) in the colour, off-odour, overall odour and overall quality in contrast to the control samples were reported for the samples prepared with chitosan, regardless of source. Control burgers and burgers with either chitosan were acceptable during the 16 days of chilled storage. The positive impact of chitosan on the sensory properties of meat products has been extensively described in the literature, with the sensory attributes of different meat products treated with chitosan (coating, film, direct addition) maintained for longer periods over the course of storage compared to controls [53,54,107,112]. Moreover, panellists were not able to distinguish pork loins treated with chitosan solution (1%) packed under vacuum from untreated samples in a triangle test until day 14 of chilled storage [128]. Nevertheless, Hautrive et al. [105] reported a negative impact of high concentrations of chitosan (4%) on the colour, aroma and overall acceptability of beef burgers when it was used as fat replacer.

The beef burgers with cranberry extract (2.5%) had a dark-purple colour, and no off-odours were observed, whilst the overall odour was not significantly different (*p* > 0.05) compared to the control. The overall quality of the burgers was marginally above the minimum threshold, and it was significantly lower (*p* < 0.05) than the untreated burgers throughout chilled storage. In other studies where sensory analysis in cooked pork and beef burgers with 2.5% cranberry was performed, slightly better odour, colour and overall acceptability of the product was reported [38,110]. Therefore, a future sensory study with cooked burgers might also provide similar findings.

The addition of 0.4% carvacrol to burgers clearly negatively impacted the odour and the overall quality of the burgers. Mixed results were noted in the study of Hulankova et al. [111] with raw and cooked burgers containing 0.2% oregano EO (72% carvacrol). The burgers had better scores than the control samples in the full course of storage, but they were above the acceptability threshold only for the first 6 days of it.

## 5. Conclusions

*L. monocytogenes* is a common pathogen found on beef carcases and meat, and it is well recognised that if beef burgers are consumed rare or undercooked the risk from the pathogen in the final product is increased. Hence, the meat industry must focus on supporting product safety as well as messaging about safe cooking practices. At the same time, consumers demand ‘clean label’ products free of synthetic preservatives, and food industries try to fulfil their expectations. As shown in the present study, the use of natural anti-microbials could be a natural alternative to enhance the safety of beef burgers stored under vacuum at chilled storage. Determination of the concentration needed to achieve a noteworthy microbial reduction in the beef burgers must be balanced against any negative impact on the organoleptic properties of the product. Chitosan, regardless of source, demonstrated good potential for the control of *L. monocytogenes* in meat products. The use of chitosan from mushroom also meets the green and circular bioeconomy agenda, being a valorised by-product from mushroom production. It also avoids the potential risk of allergenicity, associated with chitosan from the shells of crustaceans. Further research is warranted with a full eating quality assessment, impact on other pathogenic bacteria, shelf-life, and application to different types of meat products, including RTE meats.

## Figures and Tables

**Table 1:** Impact of different concentrations of natural anti-microbial agents on *L. monocytogenes* (direct plating method) in vacuum packed beef burgers during chilled storage.

		*L. monocytogenes* Counts (log_10_ CFU g^−1^) on Storage Day *				
Anti-Microbial Agent	Concentration (%)	0 d	4 d	8 d	12 d	16 d
Chitosan (Shrimp)	0	3.78 ± 0.10 ^cA1^	3.79 ± 0.05 ^dA1^	3.70 ± 0.05 ^dA1^	3.61 ± 0.21 ^dA1^	3.61 ± 0.23 ^dA1^
	0.313	3.59 ± 0.11 ^bcAB1^	3.60 ± 0.06 ^cdB1^	3.50 ± 0.07 ^cdAB1^	3.37 ± 0.15 ^cdAB1^	3.34 ± 0.12 ^cA1^
	0.625	3.49 ± 0.11 ^abB1^	3.47 ± 0.12 ^cB12^	3.34 ± 0.07 ^cAB1^	3.28 ± 0.07 ^cAB1^	3.13 ± 0.08 ^cA1^
	1.25	3.39 ± 0.05 ^abC1^	3.05 ± 0.18 ^bB1^	2.85 ± 0.10 ^bAB1^	2.84 ± 0.22 ^bAB1^	2.62 ± 0.13 ^bA1^
	2.5	3.24 ± 0.16 ^aC1^	2.70 ± 0.18 ^aB1^	2.12 ± 0.15 ^aA1^	2.15 ± 0.17 ^aA1^	2.12 ± 0.15 ^aA1^
Chitosan (Mushroom)	0	3.72 ± 0.03 ^bB1^	3.75 ± 0.07 ^dB1^	3.66 ± 0.08 ^dAB1^	3.58 ± 0.04 ^dAB1^	3.42 ± 0.03 ^dA1^
	0.313	3.40 ± 0.14 ^aB2^	3.38 ± 0.09 ^cB2^	3.22 ± 0.08 ^cAB2^	3.16 ± 0.15 ^cAB2^	2.99 ± 0.12 ^cA2^
	0.625	3.23 ± 0.08 ^aC2^	3.29 ± 0.03 ^bcC1^	3.09 ± 0.06 ^cBC2^	2.79 ± 0.20 ^bAB2^	2.87 ± 0.06 ^cA2^
	1.25	3.22 ± 0.15 ^aC1^	3.10 ± 0.21 ^bC1^	2.75 ± 0.06 ^bB1^	2.61 ± 0.23 ^bAB2^	2.47 ± 0.22 ^bA1^
	2.5	3.19 ± 0.16 ^aD1^	2.66 ± 0.16 ^aC1^	2.00 ± 0.35 ^aB1^	1.50 ± 0.34 ^aA2^	1.52 ± 0.35 ^aA2^
Cranberry extract	0	3.63 ± 0.04 ^aA1^	3.69 ± 0.08 ^bA1^	3.69 ± 0.05 ^bA1^	3.59 ± 0.05 ^cA1^	3.68 ± 0.09 ^bA1^
	0.625	3.65 ± 0.11 ^aB1^	3.57 ± 0.14 ^bB2^	3.59 ± 0.05 ^bB3^	3.24 ± 0.17 ^abA1^	3.17 ± 0.05 ^aA1^
	1.25	3.64 ± 0.04 ^aB2^	3.47 ± 0.13 ^abB2^	3.51 ± 0.09 ^abB2^	3.40 ± 0.13 ^bcB3^	3.09 ± 0.21 ^aA2^
	2.5	3.49 ± 0.16 ^aC2^	3.24 ± 0.21 ^aABC2^	3.32 ± 0.23 ^aAB2^	3.15 ± 0.15 ^aAB3^	3.07 ± 0.21 ^aA3^
Carvacrol	0	3.71 ± 0.06 ^cA^	3.67 ± 0.09 ^cA^	3.70 ± 0.13 ^cA^	3.54 ± 0.33 ^bA^	3.49 ± 0.13 ^bA^
	0.1	3.60 ± 0.08 ^bcA^	3.57 ± 0.13 ^bcA^	3.54 ± 0.09 ^bcA^	3.42 ± 0.20 ^bA^	3.41 ± 0.21 ^bA^
	0.2	3.39 ± 0.14 ^bA^	3.40 ± 0.06 ^bA^	3.35 ± 0.09 ^bA^	3.37 ± 0.18 ^bA^	3.29 ± 0.03 ^bA^
	0.4	3.06 ± 0.11 ^aA^	2.99 ± 0.06 ^aA^	2.94 ± 0.14 ^aA^	2.92 ± 0.05 ^aA^	2.86 ± 0.07 ^aA^
Thyme EO	0	3.74 ± 0.02 ^aA^	3.78 ± 0.07 ^aA^	3.76 ± 0.02 ^aA^	3.70 ± 0.04 ^aA^	3.63 ± 0.07 ^bA^
	0.125	3.76 ± 0.05 ^aA^	3.73 ± 0.07 ^aA^	3.71 ± 0.03 ^aA^	3.68 ± 0.07 ^aA^	3.57 ± 0.10 ^abA^
	0.25	3.74 ± 0.06 ^aB^	3.70 ± 0.05 ^aB^	3.60 ± 0.05 ^aAB^	3.56 ± 0.04 ^aAB^	3.42 ± 0.06 ^abA^
	0.5	3.73 ± 0.06 ^aB^	3.65 ± 0.07 ^aB^	3.57 ± 0.07 ^aAB^	3.53 ± 0.11 ^aAB^	3.36 ± 0.12 ^aA^
Hop extract	0	3.77 ± 0.03 ^aA^	3.75 ± 0.06 ^aA^	3.78 ± 0.05 ^aA^	3.68 ± 0.19 ^aA^	3.55 ± 0.18 ^aA^
	0.1	3.80 ± 0.02 ^aB^	3.76 ± 0.06 ^aB^	3.78 ± 0.05 ^aB^	3.68 ± 0.08 ^aAB^	3.48 ± 0.20 ^aA^
	0.2	3.76 ± 0.07 ^aA^	3.78 ± 0.07 ^aA^	3.76 ± 0.08 ^aA^	3.71 ± 0.04 ^aA^	3.72 ± 0.07 ^aA^
	0.4	3.71 ± 0.06 ^aA^	3.57 ± 0.12 ^aA^	3.69 ± 0.03 ^aA^	3.56 ± 0.15 ^aA^	3.52 ± 0.20 ^aA^

* Mean values ± SD. Within the same day of storage for each anti-microbial agent, means with different lowercase letters (a–d) are significantly different (*p* ≤ 0.05). Means in the same row with different capital letters (A–D) are significantly different (*p* ≤ 0.05). Within the same day of storage and at the same concentration for chitosan from shrimp, chitosan from mushroom and cranberry extract, means with different numbers (1–3) are significantly different (*p* ≤ 0.05).

**Table 2:** Impact of different concentrations of natural anti-microbial agents on *L. monocytogenes* (overlay method, allowing injured cell recovery) in vacuum packed beef burgers during chilled storage.

		*L. monocytogenes* Counts (log_10_ CFU g^−1^) on Storage Day *				
Anti-Microbial Agent	Concentration (%)	0 d	4 d	8 d	12 d	16 d
Chitosan (Shrimp)	0	3.84 ± 0.07 ^bA1^	3.77 ± 0.04 ^dA1^	3.71 ± 0.04 ^dA1^	3.64 ± 0.16 ^dA1^	3.67 ± 0.19 ^cA1^
	0.313	3.52 ± 0.14 ^aA1^	3.52 ± 0.09 ^cdA1^	3.52 ± 0.09 ^cdA1^	3.25 ± 0.10 ^cA1^	3.27 ± 0.15 ^bA1^
	0.625	3.45 ± 0.09 ^aB1^	3.36 ± 0.16 ^cAB1^	3.28 ± 0.09 ^cAB1^	3.22 ± 0.17 ^cAB1^	3.13 ± 0.06 ^bA1^
	1.25	3.30 ± 0.10 ^aB1^	2.91 ± 0.21 ^bA1^	2.83 ± 0.07 ^bA1^	2.80 ± 0.16 ^bA1^	2.66 ± 0.17 ^aA1^
	2.5	3.24 ± 0.09 ^aD1^	2.59 ± 0.19 ^aC1^	2.04 ± 0.26 ^aB1^	1.46 ± 1.11 ^aA1^	2.46 ± 0.31 ^aC1^
Chitosan (Mushroom)	0	3.71 ± 0.07 ^bB1^	3.77 ± 0.10 ^dAB1^	3.67 ± 0.03 ^dAB1^	3.60 ± 0.05 ^dAB1^	3.42 ± 0.05 ^dA1^
	0.313	3.41 ± 0.10 ^aC1^	3.36 ± 0.07 ^cBC1^	3.15 ± 0.09 ^cABC2^	3.08 ± 0.07 ^cAB1^	3.01 ± 0.13 ^cA2^
	0.625	3.28 ± 0.06 ^aB1^	3.16 ± 0.03 ^bcB1^	3.03 ± 0.11 ^bcAB1^	2.74 ± 0.21 ^bA2^	2.75 ± 0.11 ^cA2^
	1.25	3.24 ± 0.12 ^aC1^	3.02 ± 0.02 ^bBC1^	2.79 ± 0.10 ^bB1^	2.45 ± 0.05 ^bA2^	2.38 ± 0.17 ^bB2^
	2.5	3.18 ± 0.21 ^aD1^	2.59 ± 0.15 ^aC1^	2.01 ± 0.23 ^aB1^	< 1.00 ^aB2^	1.87 ± 0.35 ^aA2^
Cranberry extract	0	3.79 ± 0.08 ^aA1^	3.72 ± 0.07 ^aA1^	3.74 ± 0.04 ^aA1^	3.65 ± 0.06 ^bA1^	3.68 ± 0.09 ^bA1^
	0.625	3.77 ± 0.04 ^aB2^	3.64 ± 0.01 ^aB2^	3.59 ± 0.08 ^aB2^	3.27 ± 0.14 ^aA1^	3.22 ± 0.11 ^aA1^
	1.25	3.66 ± 0.04 ^aB2^	3.63 ± 0.04 ^aB2^	3.55 ± 0.04 ^aB2^	3.46 ± 0.16 ^abAB3^	3.27 ± 0.24 ^aA3^
	2.5	3.57 ± 0.10 ^aB2^	3.44 ± 0.10 ^aAB2^	3.46 ± 0.18 ^aAB2^	3.26 ± 0.14 ^aA3^	3.29 ± 0.08 ^aAB3^
Carvacrol	0	3.80 ± 0.04 ^cA^	3.80 ± 0.03 ^cA^	3.71 ± 0.17 ^bA^	3.69 ± 0.23 ^bA^	3.55 ± 0.13 ^bA^
	0.1	3.74 ± 0.07 ^bcA^	3.71 ± 0.04 ^bcA^	3.61 ± 0.07 ^bA^	3.52 ± 0.23 ^bA^	3.53 ± 018 ^bA^
	0.2	3.51 ± 0.02 ^abA^	3.49 ± 0.07 ^bA^	3.48 ± 0.12 ^bA^	3.41 ± 0.18 ^bA^	3.49 ± 0.13 ^bA^
	0.4	3.33 ± 0.11 ^aB^	3.18 ± 0.13 ^aAB^	3.00 ± 0.17 ^aA^	2.95 ± 0.13 ^aA^	2.94 ± 0.09 ^aA^
Thyme EO	0	3.81 ± 0.03 ^aA^	3.78 ± 0.05 ^aA^	3.77 ± 0.06 ^aA^	3.73 ± 0.04 ^aA^	3.68 ± 0.11 ^aA^
	0.125	3.81 ± 0.05 ^aA^	3.78 ± 0.07 ^aA^	3.76 ± 0.06 ^aA^	3.69 ± 0.07 ^aA^	3.61 ± 0.09 ^aA^
	0.25	3.76 ± 0.03 ^aB^	3.77 ± 0.03 ^aB^	3.71 ± 0.03 ^aAB^	3.61 ± 0.05 ^aAB^	3.42 ± 0.09 ^aA^
	0.5	3.74 ± 0.03 ^aB^	3.69 ± 0.09 ^aAB^	3.61 ± 0.04 ^aAB^	3.61 ± 0.07 ^aAB^	3.41 ± 0.12 ^aA^
Hop extract	0	3.82 ± 0.05 ^aA^	3.74 ± 0.04 ^aA^	3.82 ± 0.04 ^aA^	3.76 ± 0.15 ^aA^	3.63 ± 0.20 ^abA^
	0.1	3.81 ± 0.05 ^aB^	3.84 ± 0.05 ^aB^	3.79 ± 0.06 ^aB^	3.69 ± 0.04 ^aAB^	3.47 ± 0.22 ^aA^
	0.2	3.79 ± 0.04 ^aA^	3.77 ± 0.04 ^aA^	3.80 ± 0.05 ^aA^	3.77 ± 0.03 ^aA^	3.77 ± 0.06 ^bA^
	0.4	3.83 ± 0.07 ^aA^	3.80 ± 0.03 ^aA^	3.76 ± 0.03 ^aA^	3.74 ± 0.10 ^aA^	3.74 ± 0.15 ^abA^

* Mean values ± SD. Within the same day of storage for each anti-microbial agent, means with different lowercase letters (a–d) are significantly different (*p* ≤ 0.05). Means in the same row with different capital letters (A–D) are significantly different (*p* ≤ 0.05). Within the same day of storage and within the same concentration for chitosan from shrimp, chitosan from mushroom and cranberry extract, means with different numbers (1–3) are significantly different (*p* ≤ 0.05).

**Table 3:** Impact of natural anti-microbial agents on the pH of vacuum packed beef burgers during chilled storage.

Treatment	Storage Day		
	0 d	8 d	16 d
Control	5.68 ± 0.02 ^bB 1^	5.65 ± 0.03 ^bAB^	5.58 ± 0.09 ^bA^
Chitosan (Shrimp) (2.5%)	6.55 ± 0.09 ^cB^	6.57 ± 0.08 ^cB^	6.40 ± 0.07 ^cA^
Chitosan (Mushroom) (2.5%)	6.62 ± 0.04 ^cB^	6.68 ± 0.02 ^cB^	6.49 ± 0.12 ^cA^
Cranberry extract (2.5%)	5.31 ± 0.02 ^aA^	5.27 ± 0.02 ^aA^	5.22 ± 0.11 ^aA^
Carvacrol (0.4%)	5.66 ± 0.02 ^bA^	5.65 ± 0.03 ^bA^	5.60 ± 0.09 ^bA^

^1^ Within the same day of storage, means with different lowercase letters (a–c) are significantly different (*p* ≤ 0.05). Means in the same row with different capital letters (A,B) are significantly different (*p* ≤ 0.05).

**Table 4:** Impact of natural anti-microbial agents on the colour parameters L* (lightness/darkness), a* (redness/greenness) and b* (yellowness/blueness) of vacuum packed beef burgers during chilled storage.

Colour	Treatment	Storage Day		
		0 d	8 d	16 d
L*	Control	46.30 ± 1.93 ^bA 1^	46.63 ± 0.19 ^bA^	46.20 ± 2.28 ^bA^
	Chitosan (Shrimp) (2.5%)	45.21 ± 1.16 ^bA^	48.71 ± 2.46 ^bcB^	49.04 ± 3.32 ^bcB^
	Chitosan (Mushroom) (2.5%)	45.63 ± 2.44 ^bA^	46.48 ± 1.04 ^bA^	46.53 ± 1.18 ^bcA^
	Cranberry extract (2.5%)	31.25 ± 0.71 ^aA^	33.09 ± 1.58 ^aAB^	33.83 ± 0.64 ^aB^
	Carvacrol (0.4%)	47.82 ± 1.51 ^bA^	49.66 ± 1.82 ^cA^	49.39 ± 2.28 ^cA^
a*	Control	11.81 ± 3.32 ^bA^	10.91 ± 0.31 ^aA^	10.76 ± 0.84 ^bA^
	Chitosan (Shrimp) (2.5%)	8.67 ± 0.36 ^aA^	9.33 ± 0.58 ^aA^	9.98 ± 1.21 ^abA^
	Chitosan (Mushroom) (2.5%)	9.05 ± 0.53 ^aA^	9.89 ± 0.51 ^aA^	10.42 ± 0.57 ^abA^
	Cranberry extract (2.5%)	9.20 ± 0.73 ^aA^	9.02 ± 0.18 ^aA^	8.64 ± 0.57 ^aA^
	Carvacrol (0.4%)	10.30 ± 2.12 ^abA^	10.41 ± 0.38 ^aA^	10.32 ± 1 ^abA^
b*	Control	14.40 ± 2.07 ^cB^	11.90 ± 0.21 ^bA^	11.98 ± 0.81 ^bA^
	Chitosan (Shrimp) (2.5%)	12.80 ± 0.87 ^bA^	13.36 ± 1.14 ^bA^	13.46 ± 1.46 ^bA^
	Chitosan (Mushroom) (2.5%)	12.65 ± 1.42 ^bA^	12.46 ± 0.51 ^bA^	12.95 ± 0.60 ^bA^
	Cranberry extract (2.5%)	0.00 ± 0.30 ^aA^	0.25 ± 0.33 ^aA^	0.74 ± 0.19 ^aB^
	Carvacrol (0.4%)	14.44 ± 1.01 ^cA^	13.10 ± 0.85 ^bA^	13.56 ± 0.83 ^bA^

^1^ Within the same day of storage for each colour parameter, means with different lowercase letters (a–c) are significantly different (*p* ≤ 0.05). Means in the same row with different capital letters (A,B) are significantly different (*p* ≤ 0.05).

**Table 5:** Impact of natural anti-microbial agents on visual colour, off-odour, overall dour and overall quality of vacuum packed beef burgers during chilled storage (at 0 min after opening the packaging).

Attribute	Treatment	Storage Day		
		0 d	8 d	16 d
Visual colour ^2^	Control	3.15 ^aB 1^ [2.69–3.61]	2.08 ^aA^ [1.66–2.5]	2.58 ^aAB^ [2.16–3]
	Chitosan (Shrimp) (2.5%)	3.20 ^aA^ [2.74–3.66]	2.58 ^aA^ [2.16–3]	2.83 ^aA^ [2.41–3.25]
	Chitosan (Mushroom) (2.5%)	3.25 ^aB^ [2.79–3.71]	2.5 ^aA^ [2.08–2.92]	2.71 ^aAB^ [2.29–3.13]
	Cranberry extract (2.5%)	5.95 ^bA^ [5.49–6] ^6^	6 ^bA^ [5.58–6] ^6^	6 ^bA^ [5.58–6] ^6^
	Carvacrol (0.4%)	3.25 ^aB^ [2.79–3.71]	2.38 ^aA^ [1.96–2.79]	2.88 ^aAB^ [2.46–3.29]
Off-odour ^3^	Control	1.25 ^aA^ [1–1.58] ^6^	1.63 ^aA^ [1.33–1.92]	1.63 ^abA^ [1.33–1.92]
	Chitosan (Shrimp) (2.5%)	1.3 ^aA^ [1–1.63] ^6^	1.29 ^aA^ [1–1.59] ^6^	2 ^bB^ [1.7–2.3]
	Chitosan (Mushroom) (2.5%)	1.55 ^aA^ [1.22–1.88]	1.29 ^aA^ [1–1.59] ^6^	1.46 ^abA^ [1.16–1.76]
	Cranberry extract (2.5%)	1.45 ^aA^ [1.12–1.78]	1.29 ^aA^ [1–1.59] ^5^	1.21 ^aA^ [1–1.51] ^5^
	Carvacrol (0.4%)	1.5 ^aA^ [1.17–1.83]	1.25 ^aA^ [1–1.55] ^6^	1.33 ^aA^ [1.03–1.63]
Overall odour ^4^	Control	3.55 ^aA^ [2.61–4.49]	3.79 ^aA^ [2.94–4.65]	4.04 ^aA^ [3.19–4.9]
	Chitosan (Shrimp) (2.5%)	3.35 ^aA^ [2.41–4.29]	4.04 ^abA^ [3.19–4.9]	4.17 ^aA^ [3.31–5.02]
	Chitosan (Mushroom) (2.5%)	3.6 ^aA^ [2.66–4.54]	4 ^aA^ [3.14–4.86]	4.42 ^aA^ [3.56–5.27]
	Cranberry extract (2.5%)	5.1 ^abA^ [4.16–6.04]	4.67 ^abA^ [3.81–5.52]	4.21 ^aA^ [3.35–5.06]
	Carvacrol (0.4%)	5.6 ^bA^ [4.66–6.54]	5.71 ^bA^ [4.85–6.56]	5.29 ^aA^ [4.44–6.15]
Overall quality ^5^	Control	7.15 ^bA^ [6.66–7.64]	7.21 ^bA^ [6.76–7.66]	7.17 ^bA^ [6.72–7.62]
	Chitosan (Shrimp) (2.5%)	7.01 ^bA^ [6.61–7.59]	7.46 ^bA^ [7.01–7.91]	6.71 ^bA^ [6.26–7.16]
	Chitosan (Mushroom) (2.5%)	7.3 ^bA^ [6.81–7.79]	7.5 ^bA^ [7.05–7.95]	7.42 ^bA^ [6.97–7.87]
	Cranberry extract (2.5%)	5.35 ^aA^ [4.86–5.84]	5.42 ^aA^ [4.97–5.87]	5.42 ^aA^ [4.97–5.87]
	Carvacrol (0.4%)	5.55 ^aA^ [5.06–6.04]	6.04 ^aA^ [5.59–6.49]	5.79 ^aA^ [5.34–6.24]

^1^ Mean scores with 95% confidence intervals. Within the same day of storage, for each attribute, mean scores with different lowercase letters (a,b) are significantly different (*p* ≤ 0.05). Mean scores in the same row with different capital letters (A,B) are significantly different (*p* ≤ 0.05). ^2^ Visual colour: 1 = Bright purple-red; 2 = Dull purple-red; 3 = Slightly brownish-red; 4 = Moderately brownish-red; 5 = Brown; 6 = Dark purple. ^3^ Off-odour: 1 = None; 2 = Trace, not sure; 3 = Slight; 4 = Mild; 5 = Moderate; 6 = Definite; 7 = Strong; 8 = Very strong. ^4^ Overall odour: 1 = None; 2 = Trace, not sure; 3 = Slight; 4 = Mild; 5 = Moderate; 6 = Definite; 7 = Strong; 8 = Very strong. ^5^ Overall quality: 1, 2 = Reject; 3, 4, 5 = Unacceptable; 6, 7, 8 = Acceptable; 9, 10 = Match. ^6^ When the lower confidence interval falls below the minimum score or the upper confidence interval exceeds the maximum score, the minimum and maximum scores are included in the interval, respectively.

**Table 6:** Impact of natural anti-microbial agents on visual colour, off-odour, overall odour and overall quality of vacuum packed beef burgers during chilled storage (at 30 min after opening the packaging).

Attribute	Treatment	Storage Day		
		0 d	8 d	16 d
Visual colour ^2^	Control	3.15 ^Ab 1^ [2.66–3.64]	2.25 ^aA^ [1.81–2.69]	2.42 ^aAB^ [1.97–2.86]
	Chitosan (Shrimp) (2.5%)	2.9 ^aA^ [2.41–3.39]	2.42 ^aA^ [1.97–2.86]	2.75 ^aA^ [2.31–3.19]
	Chitosan (Mushroom) (2.5%)	2.85 ^aA^ [2.36–3.34]	2.21 ^aA^ [1.76–2.65]	2.29 ^aA^ [1.85–2.74]
	Cranberry extract (2.5%)	5.8 ^bA^ [5.31–6] ^6^	6 ^bA^ [5.56–6] ^6^	5.75 ^bA^ [5.31–6] ^6^
	Carvacrol (0.4%)	3.2 ^aB^ [2.71–3.69]	2.58 ^aAB^ [2.14–3.03]	2.29 ^aA^ [1.85–2.74]
Off-odour ^3^	Control	1.15 ^aA^ [1–1.45] ^6^	1.67 ^aB^ [1.39–1.94]	1.38 ^abAB^ [1.10–1.65]
	Chitosan (Shrimp) (2.5%)	1.25 ^aA^ [1–1.55] ^6^	1.42 ^aAB^ [1.14–1.69]	1.75 ^bB^ [1.47–2.03]
	Chitosan (Mushroom) (2.5%)	1.4 ^aA^ [1.10–1.70]	1.33 ^aA^ [1.06–1.61]	1.5 ^abA^ [1.22–1.78]
	Cranberry extract (2.5%)	1.25 ^aA^ [1–1.55] ^6^	1.17 ^aA^ [1–1.44] ^6^	1 ^aA^ [1–1.28] ^6^
	Carvacrol (0.4%)	1.55 ^aA^ [1–1.44] ^6^	1.17 ^aA^ [1–1.55] ^6^	1.21 ^abA^ [1–1.49] ^6^
Overall odour ^4^	Control	3.35 ^aA^ [2.46–4.24]	3.96 ^aA^ [3.15–4.77]	3.71 ^aA^ [2.90–4.52]
	Chitosan (Shrimp) (2.5%)	3.5 ^aA^ [2.61–4.39]	3.96 ^aA^ [3.15–4.77]	3.88 ^aA^ [3.06–4.69]
	Chitosan (Mushroom) (2.5%)	3.55 ^aA^ [2.66–4.44]	3.75 ^aA^ [2.94–4.56]	4.33 ^aA^ [3.52–5.14]
	Cranberry extract (2.5%)	5 ^abA^ [4.11–5.89]	4.75 ^aA^ [3.94–5.56]	4.17 ^aA^ [3.36–4.98]
	Carvacrol (0.4%)	5.35 ^bA^ [4.46–6.24]	5.33 ^aA^ [4.52–6.14]	5 ^aA^ [4.19–5.81]
Overall quality ^5^	Control	7.3 ^bA^ [6.84–7.76]	7.25 ^bA^ [6.83–7.67]	6.96 ^cA^ [6.54–7.38]
	Chitosan (Shrimp) (2.5%)	7.15 ^bAB^ [6.69–7.61]	7.46 ^bB^ [7.04–7.88]	6.58 ^bcA^ [6.17–7]
	Chitosan (Mushroom) (2.5%)	7.2 ^bA^ [6.74–7.66]	7.54 ^bA^ [7.12–7.96]	7.13 ^cA^ [6.71–7.54]
	Cranberry extract (2.5%)	5.1 ^aA^ [4.64–5.56]	5.46 ^aA^ [5.04–5.88]	5.67 ^aA^ [5.25–6.08]
	Carvacrol (0.4%)	5.35 ^aA^ [4.89–5.81]	5.83 ^aA^ [5.42–6.25]	5.79 ^abA^ [5.37–6.21]

^1^ Mean scores with 95% confidence intervals. Within the same day of storage, for each attribute, mean scores with different lowercase letters (a–c) are significantly different (*p* ≤ 0.05). Mean scores in the same row with different capital letters (A,B) are significantly different (*p* ≤ 0.05). ^2^ Visual colour: 1 = Bright purple-red; 2 = Dull purple-red; 3 = Slightly brownish-red; 4 = Moderately brownish-red; 5 = Brown; 6 = Dark purple. ^3^ Off-odour: 1 = None; 2 = Trace, not sure; 3 = Slight; 4 = Mild; 5 = Moderate; 6 = Definite; 7 = Strong; 8 = Very strong. ^4^ Overall odour: 1 = None; 2 = Trace, not sure; 3 = Slight; 4 = Mild; 5 = Moderate; 6 = Definite; 7 = Strong; 8 = Very strong. ^5^ Overall quality: 1, 2 = Reject; 3, 4, 5 = Unacceptable; 6, 7, 8 = Acceptable; 9, 10 = Match. ^6^ When the lower confidence interval falls below the minimum score or the upper confidence interval exceeds the maximum score, the minimum and maximum scores are included in the interval, respectively.

## Data Availability

The original contributions presented in this study are included in the article/Supplementary Materials. Further inquiries can be directed to the corresponding author.

## Supplementary Materials

The following supporting information can be downloaded at https://www.mdpi.com/article/10.3390/microorganisms13040910/s1, Table S1: Minimum Inhibitory and Bactericidal Concentrations (MIC and MBC) of a range of natural anti-microbial agents against *L. monocytogenes* under different conditions of growth; Table S2: Effect sizes (η^2^) for the impact of natural anti-microbial agents on the visual colour, off-odour, overall odour and overall quality of vacuum packed beef burgers during chilled storage (at 0 min after opening the packaging); Table S3: Effect sizes (η^2^) for the impact of natural anti-microbial agents on the visual colour, off-odour, overall odour and overall quality of vacuum packed beef burgers during chilled storage (at 30 min after opening the packaging).

## References

[1] Sauders B.D., Overdevest J., Fortes E., Windham K., Schukken Y., Lembo A., Wiedmann M. (2012). Diversity of *Listeria* species in urban and natural environments. Appl. Environ. Microbiol..

[2] Nightingale K.K., Schukken Y.H., Nightingale C.R., Fortes E.D., Ho A.J., Her Z., Grohn Y.T., McDonough P.L., Wiedmann M. (2004). Ecology and transmission of *Listeria monocytogenes* infecting ruminants and in the farm environment. Appl. Environ. Microbiol..

[3] Vivant A.L., Garmyn D., Piveteau P. (2013). *Listeria monocytogenes*, a down-to-earth pathogen. Front. Cell Infect. Microbiol..

[4] Weis J., Seeliger H.P.R. (1975). Incidence of *Listeria monocytogenes* in Nature. Appl. Microbiol..

[5] Linke K., Ruckerl I., Brugger K., Karpiskova R., Walland J., Muri-Klinger S., Tichy A., Wagner M., Stessl B. (2014). Reservoirs of *Listeria* species in three environmental ecosystems. Appl. Environ. Microbiol..

[6] Olier M., Pierre F., Tre J.-P.L., Divies C., Rousset A., Guzzo J. (2002). Assessment of the pathogenic potential of two *Listeria monocytogenes* human faecal carriage isolates. Microbiology.

[7] Watkins J., Sleath K.P. (1981). Isolation and Enumeration of *Listeria monocytogenes* from Sewage, Sewage Sludge and River Water. J. Appl. Bacteriol..

[8] Matle I., Mbatha K.R., Madoroba E. (2020). A review of *Listeria monocytogenes* from meat and meat products: Epidemiology, virulence factors, antimicrobial resistance and diagnosis. Onderstepoort J. Vet. Res..

[9] Ferreira V., Wiedmann M., Teixeira P., Stasiewicz M.J. (2014). *Listeria monocytogenes* persistence in food-associated environments: Epidemiology, strain characteristics, and implications for public health. J. Food Prot..

[10] Zhu Q., Gooneratne R., Hussain M.A. (2017). *Listeria monocytogenes* in Fresh Produce: Outbreaks, Prevalence and Contamination Levels. Foods.

[11] Khen B.K., Lynch O.A., Carroll J., McDowell D.A., Duffy G. (2015). Occurrence, antibiotic resistance and molecular characterization of *Listeria monocytogenes* in the beef chain in the Republic of Ireland. Zoonoses Public Health.

[12] Rhoades J.R., Duffy G., Koutsoumanis K. (2009). Prevalence and concentration of verocytotoxigenic *Escherichia coli*, *Salmonella enterica* and *Listeria monocytogenes* in the beef production chain: A review. Food Microbiol..

[13] Demaitre N., De Reu K., Haegeman A., Schaumont D., De Zutter L., Geeraerd A., Rasschaert G. (2021). Study of the transfer of *Listeria monocytogenes* during the slaughter of cattle using molecular typing. Meat Sci..

[14] Wieczorek K., Dmowska K., Osek J. (2012). Prevalence, characterization, and antimicrobial resistance of *Listeria monocytogenes* isolates from bovine hides and carcasses. Appl. Environ. Microbiol..

[15] CAC (2007). Guidelines on the Application of General Principles of Food Hygiene to the Control of *Listeria monocytogenes* in Foods. CAC/GL 61—2007. https://www.nicd.ac.za/wp-content/uploads/2018/05/Guidelines_on_the_Application_of_General_Principles_of_Food_Hygiene_to_the_Control_of_Listeria_Monocytogenes_in_Foods_CAC_GL_61-2007.pdf.

[16] Mintel (2016). Gourmet Burger Sector Report—UK—January 2016. https://www.food.gov.uk/sites/default/files/media/document/fsa10029finalreport.pdf.

[17] Bogard A.K., Fuller C.C., Radke V., Selman C.A., Smith K.E. (2013). Ground beef handling and cooking practices in restaurants in eight States. J. Food Prot..

[18] Smith A.M., Tau N.P., Smouse S.L., Allam M., Ismail A., Ramalwa N.R., Disenyeng B., Ngomane M., Thomas J. (2019). Outbreak of *Listeria monocytogenes* in South Africa, 2017–2018: Laboratory Activities and Experiences Associated with Whole-Genome Sequencing Analysis of Isolates. Foodborne Pathog. Dis..

[19] WHO Listeriosis—South Africa. https://www.who.int/emergencies/disease-outbreak-news/item/28-march-2018-listeriosis-south-africa-en.

[20] ECDC Rapid Outbreak Assessment: Multi-Country Outbreak of Listeria monocytogenes Sequence Type 6 Infections Linked to Ready-to-Eat Meat Products. https://www.ecdc.europa.eu/en/publications-data/rapid-outbreak-assessment-multi-country-outbreak-listeria-monocytogenes-sequence.

[21] Sung S.-Y., Sin L.T., Tee T.-T., Bee S.-T., Rahmat A.R., Rahman W.A.W.A., Tan A.-C., Vikhraman M. (2013). Antimicrobial agents for food packaging applications. Trends Food Sci. Technol..

[22] Amit S.K., Uddin M.M., Rahman R., Islam S.M.R., Khan M.S. (2017). A review on mechanisms and commercial aspects of food preservation and processing. Agric. Food Secur..

[23] Quintavalla S., Vicini L. (2002). Antimicrobial food packaging in meat industry. Meat Sci..

[24] Lucera A., Costa C., Conte A., Del Nobile M.A. (2012). Food applications of natural antimicrobial compounds. Front. Microbiol..

[25] Delgado-Pando G., Ekonomou S.I., Stratakos A.C., Pintado T. (2021). Clean Label Alternatives in Meat Products. Foods.

[26] Sbardelotto P.R.R., Balbinot-Alfaro E., da Rocha M., Alfaro A.T. (2023). Natural alternatives for processed meat: Legislation, markets, consumers, opportunities and challenges. Crit. Rev. Food Sci. Nutr..

[27] Papadochristopoulos A., Kerry J.P., Fegan N., Burgess C.M., Duffy G. (2021). Natural Anti-Microbials for Enhanced Microbial Safety and Shelf-Life of Processed Packaged Meat. Foods.

[28] Solomakos N., Govaris A., Koidis P., Botsoglou N. (2008). The antimicrobial effect of thyme essential oil, nisin, and their combination against *Listeria monocytogenes* in minced beef during refrigerated storage. Food Microbiol..

[29] Yemis G.P., Candogan K. (2017). Antibacterial activity of soy edible coatings incorporated with thyme and oregano essential oils on beef against pathogenic bacteria. Food Sci. Biotechnol..

[30] Veldhuizen E.J., Creutzberg T.O., Burt S.A., Haagsman H.P. (2007). Low temperature and binding to food components inhibit the antibacterial activity of carvacrol against *Listeria monocytogenes* in steak tartare. J. Food Prot..

[31] Somrani M., Debbabi H., Palop A. (2022). Antibacterial and antibiofilm activity of essential oil of clove against *Listeria monocytogenes* and *Salmonella Enteritidis*. Food Sci. Technol. Int..

[32] Khaleque M.A., Keya C.A., Hasan K.N., Hoque M.M., Inatsu Y., Bari M.L. (2016). Use of cloves and cinnamon essential oil to inactivate *Listeria monocytogenes* in ground beef at freezing and refrigeration temperatures. LWT Food Sci. Technol..

[33] Raeisi M., Tabaraei A., Hashemi M., Behnampour N. (2016). Effect of sodium alginate coating incorporated with nisin, *Cinnamomum zeylanicum*, and rosemary essential oils on microbial quality of chicken meat and fate of *Listeria monocytogenes* during refrigeration. Int. J. Food Microbiol..

[34] Gouveia A.R., Alves M., de Almeida J.M.M.M., Monteiro-Silva F., González-Aguilar G., Silva J.A., Saraiva C. (2017). The Antimicrobial Effect of Essential Oils Against *Listeria monocytogenes* in *Sous vide* Cook-Chill Beef during Storage. J. Food Process. Preserv..

[35] Kramer B., Thielmann J., Hickisch A., Muranyi P., Wunderlich J., Hauser C. (2015). Antimicrobial activity of hop extracts against foodborne pathogens for meat applications. J. Appl. Microbiol..

[36] Kramer B., Mignard C., Warschat D., Gürbüz S., Aiglstorfer P., Muranyi P. (2021). Inhibition of *Listeria monocytogenes* on bologna by a beta acid rich hop extract. Food Control.

[37] Tamkute L., Gil B.M., Carballido J.R., Pukalskiene M., Venskutonis P.R. (2019). Effect of cranberry pomace extracts isolated by pressurized ethanol and water on the inhibition of food pathogenic/spoilage bacteria and the quality of pork products. Food Res. Int..

[38] Stobnicka A., Gniewosz M. (2018). Antimicrobial protection of minced pork meat with the use of Swamp Cranberry (*Vaccinium oxycoccos* L.) fruit and pomace extracts. J. Food Sci. Technol..

[39] Qiu X., Wu V.C.H. (2007). Evaluation of *Escherichia coli* O157:H7, *Listeria monocytogenes*, *Salmonella* Typhimurium and *Staphylococcus aureus* in ground beef with cranberry concentrate by thin agar layer method. J. Rapid Methods Autom. Microbiol..

[40] Mohdaly A.A.A., Mahmoud A.A., Roby M.H.H., Smetanska I., Ramadan M.F. (2015). Phenolic Extract from Propolis and Bee Pollen: Composition, Antioxidant and Antibacterial Activities. J. Food Biochem..

[41] Economou V., Tsitsos A., Theodoridis A., Ambrosiadis I., Arsenos G. (2022). Effects of Chitosan Coatings on Controlling *Listeria monocytogenes* and Methicillin-Resistant *Staphylococcus aureus* in Beef and Mutton Cuts. Appl. Sci..

[42] Antoniadou D., Govaris A., Ambrosiadis I., Sergelidis D. (2019). Effect of chitosan coating on the shelf life of ready-to-eat bovine meatballs and the control of *Listeria monocytogenes* growth on their surface during refrigeration storage. J. Hellenic Vet. Med. Soc..

[43] Wang G.H. (1992). Inhibition and Inactivation of Five Species of Foodborne Pathogens by Chitosan. J. Food Prot..

[44] Hu Z.Y., Balay D., Hu Y., McMullen L.M., Ganzle M.G. (2019). Effect of chitosan, and bacteriocin—Producing *Carnobacterium maltaromaticum* on survival of *Escherichia coli* and *Salmonella Typhimurium* on beef. Int. J. Food Microbiol..

[45] Zimoch-Korzycka A., Jarmoluk A. (2015). The use of chitosan, lysozyme, and the nano-silver as antimicrobial ingredients of edible protective hydrosols applied into the surface of meat. J. Food Sci. Technol..

[46] He L., Zou L., Yang Q., Xia J., Zhou K., Zhu Y., Han X., Pu B., Hu B., Deng W. (2016). Antimicrobial Activities of Nisin, Tea Polyphenols, and Chitosan and their Combinations in Chilled Mutton. J. Food Sci..

[47] Ibanez-Peinado D., Ubeda-Manzanaro M., Martinez A., Rodrigo D. (2020). Antimicrobial effect of insect chitosan on *Salmonella Typhimurium*, *Escherichia coli* O157:H7 and *Listeria monocytogenes* survival. PLoS ONE.

[48] Chang S.H., Chen C.H., Tsai G.J. (2020). Effects of Chitosan on *Clostridium perfringens* and Application in the Preservation of Pork Sausage. Mar. Drugs.

[49] Juneja V.K., Thippareddi H., Bari L., Inatsu Y., Kawamoto S., Friedman M. (2006). Chitosan Protects Cooked Ground Beef and Turkey Against *Clostridium perfringens* Spores During Chilling. J. Food Sci..

[50] İncili G.K., Karatepe P., İlhak O.İ. (2020). Effect of chitosan and *Pediococcus acidilactici* on *E. coli* O157:H7, *Salmonella* Typhimurium and *Listeria monocytogenes* in meatballs. LWT Food Sci. Technol..

[51] Ruiz-Hernández K., Sosa-Morales M.E., Cerón-García A., Gómez-Salazar J.A. (2021). Physical, Chemical and Sensory Changes in Meat and Meat Products Induced by the Addition of Essential Oils: A Concise Review. Food Rev. Int..

[52] Xi Y., Sullivan G.A., Jackson A.L., Zhou G.H., Sebranek J.G. (2012). Effects of natural antimicrobials on inhibition of *Listeria monocytogenes* and on chemical, physical and sensory attributes of naturally-cured frankfurters. Meat Sci..

[53] Chounou N., Chouliara E., Mexis S.F., Stavros K., Georgantelis D., Kontominas M.G. (2013). Shelf life extension of ground meat stored at 4°C using chitosan and an oxygen absorber. Int. J. Food Sci. Technol..

[54] Đorđević N., Karabegović I., Cvetković D., Šojić B., Savić D., Danilović B. (2022). Assessment of Chitosan Coating Enriched with Free and Nanoencapsulated *Satureja montana* L. Essential Oil as a Novel Tool for Beef Preservation. Foods.

[55] Santiesteban-Lopez N.A., Gomez-Salazar J.A., Santos E.M., Campagnol P.C.B., Teixeira A., Lorenzo J.M., Sosa-Morales M.E., Dominguez R. (2022). Natural Antimicrobials: A Clean Label Strategy to Improve the Shelf Life and Safety of Reformulated Meat Products. Foods.

[56] Cruz-Romero M.C., Murphy T., Morris M., Cummins E., Kerry J.P. (2013). Antimicrobial activity of chitosan, organic acids and nano-sized solubilisates for potential use in smart antimicrobially-active packaging for potential food applications. Food Control.

[57] Giatrakou V., Ntzimani A., Savvaidis I.N. (2010). Effect of chitosan and thyme oil on a ready to cook chicken product. Food Microbiol..

[58] Rivas L., McDonnell M.J., Burgess C.M., O’Brien M., Navarro-Villa A., Fanning S., Duffy G. (2010). Inhibition of verocytotoxigenic *Escherichia coli* in model broth and rumen systems by carvacrol and thymol. Int. J. Food Microbiol..

[59] Burt S.A., Vlielander R., Haagsman H.P., Veldhuizen E.J. (2005). Increase in Activity of Essential Oil Components Carvacrol and Thymol against *Escherichia coli* O157:H7 by Addition of Food Stabilizers. J. Food Prot..

[60] Dygico L.K., Gahan C.G.M., Grogan H., Burgess C.M. (2021). Examining the efficacy of mushroom industry biocides on *Listeria monocytogenes* biofilm. J. Appl. Microbiol..

[61] (2008). Moisture and Fat in Meats. Microwave and Nuclear Magnetic Resonance Analysis.

[62] Busch S.V., Donnelly C.W. (1992). Development of a Repair-Enrichment Broth for Resuscitation of Heat-Injured *Listeria monocytogenes* and *Listeria innocua*. Appl. Environ. Microbiol..

[63] Moran L., Andres S., Bodas R., Prieto N., Giraldez F.J. (2012). Meat texture and antioxidant status are improved when carnosic acid is included in the diet of fattening lambs. Meat Sci..

[64] Maraschiello C., Sárraga C., García Regueiro J.A. (1999). Glutathione peroxidase activity, TBARS, and α-tocopherol in meat from chickens fed different diets. J. Agric. Food Chem..

[65] AMSA (2016). Research Guidelines for Cookery, Sensory Evaluation, and Instrumental Tenderness Measurements of Meat.

[66] King S., Gillette M., Titman D., Adams J., Ridgely M. (2002). The Sensory Quality System: A global quality control solution. Food Qual. Prefer..

[67] Kaur M., Shang H., Tamplin M., Ross T., Bowman J.P. (2017). Culture-dependent and culture-independent assessment of spoilage community growth on VP lamb meat from packaging to past end of shelf-life. Food Microbiol..

[68] Ortiz-Sola J., Vinas I., Colas-Meda P., Anguera M., Abadias M. (2020). Occurrence of selected viral and bacterial pathogens and microbiological quality of fresh and frozen strawberries sold in Spain. Int. J. Food Microbiol..

[69] Lamas A., Miranda J.M., Vazquez B., Cepeda A., Franco C.M. (2016). An Evaluation of Alternatives to Nitrites and Sulfites to Inhibit the Growth of *Salmonella enterica* and *Listeria monocytogenes* in Meat Products. Foods.

[70] Kong M., Chen X.G., Xing K., Park H.J. (2010). Antimicrobial properties of chitosan and mode of action: A state of the art review. Int. J. Food Microbiol..

[71] Yan D., Li Y., Liu Y., Li N., Zhang X., Yan C. (2021). Antimicrobial Properties of Chitosan and Chitosan Derivatives in the Treatment of Enteric Infections. Molecules.

[72] Raafat D., von Bargen K., Haas A., Sahl H.G. (2008). Insights into the mode of action of chitosan as an antibacterial compound. Appl. Environ. Microbiol..

[73] Häkkinen S.H., Kärenlampi S.O., Heinonen I.M., Mykkänen H.M., Törrönen A.R. (1999). Content of the Flavonols Quercetin, Myricetin, and Kaempferol in 25 Edible Berries. J. Agric. Food Chem..

[74] Puupponen-Pimiä R., Nohynek L., Hartmann-Schmidlin S., Kähkönen M., Heinonen M., Määttä-Riihinen K., Oksman-Caldentey K.M. (2005). Berry phenolics selectively inhibit the growth of intestinal pathogens. J. Appl. Microbiol..

[75] Jurikova T., Skrovankova S., Mlcek J., Balla S., Snopek L. (2018). Bioactive Compounds, Antioxidant Activity, and Biological Effects of European Cranberry (*Vaccinium oxycoccos*). Molecules.

[76] Bae J.Y., Seo Y.H., Oh S.W. (2022). Antibacterial activities of polyphenols against foodborne pathogens and their application as antibacterial agents. Food Sci. Biotechnol..

[77] Wu V.C.H., Qiu X., Bushway A., Harper L. (2008). Antibacterial effects of American cranberry (*Vaccinium macrocarpon*) concentrate on foodborne pathogens. LWT Food Sci. Technol..

[78] Diarra M.S., Block G., Rempel H., Oomah B.D., Harrison J., McCallum J., Boulanger S., Brouillette É., Gattuso M., Malouin F. (2013). In vitro and in vivo antibacterial activities of cranberry press cake extracts alone or in combination with β-lactams against *Staphylococcus aureus*. BMC Complement. Altern. Med..

[79] De Vincenzi M., Stammati A., De Vincenzi A., Silano M. (2004). Constituents of aromatic plants: Carvacrol. Fitoterapia.

[80] Arioli S., Montanari C., Magnani M., Tabanelli G., Patrignani F., Lanciotti R., Mora D., Gardini F. (2019). Modelling of *Listeria monocytogenes* Scott A after a mild heat treatment in the presence of thymol and carvacrol: Effects on culturability and viability. J. Food Eng..

[81] Mazzarrino G., Paparella A., Chaves-López C., Faberi A., Sergi M., Sigismondi C., Compagnone D., Serio A. (2015). *Salmonella enterica* and *Listeria monocytogenes* inactivation dynamics after treatment with selected essential oils. Food Control.

[82] Pesavento G., Calonico C., Bilia A.R., Barnabei M., Calesini F., Addona R., Mencarelli L., Carmagnini L., Di Martino M.C., Lo Nostro A. (2015). Antibacterial activity of Oregano, Rosmarinus and Thymus essential oils against *Staphylococcus aureus* and *Listeria monocytogenes* in beef meatballs. Food Control.

[83] Ultee A., Bennik M.H., Moezelaar R. (2002). The phenolic hydroxyl group of carvacrol is essential for action against the food-borne pathogen *Bacillus cereus*. Appl. Environ. Microbiol..

[84] Burt S. (2004). Essential oils: Their antibacterial properties and potential applications in foods-a review. Int. J. Food Microbiol..

[85] He Q., Zhang L., Yang Z., Ding T., Ye X., Liu D., Guo M. (2022). Antibacterial mechanisms of thyme essential oil nanoemulsions against *Escherichia coli* O157:H7 and *Staphylococcus aureus*: Alterations in membrane compositions and characteristics. Innov. Food Sci. Emerg. Technol..

[86] Tian B., Li W., Wang J., Liu Y. (2021). Functional polysaccharide-based film prepared from chitosan and beta-acids: Structural, physicochemical, and bioactive properties. Int. J. Biol. Macromol..

[87] Kong M., Chen X.-G., Xue Y.-P., Liu C.-S., Yu L.-J., Ji Q.-X., Cha D.S., Park H.J. (2008). Preparation and antibacterial activity of chitosan microshperes in a solid dispersing system. Front. Mater. Sci. China.

[88] Chien R.C., Yen M.T., Mau J.L. (2016). Antimicrobial and antitumor activities of chitosan from shiitake stipes, compared to commercial chitosan from crab shells. Carbohydr. Polym..

[89] Sogias I.A., Khutoryanskiy V.V., Williams A.C. (2010). Exploring the Factors Affecting the Solubility of Chitosan in Water. Macromol. Chem. Phys..

[90] Huq T., Khan A., Brown D., Dhayagude N., He Z., Ni Y. (2022). Sources, production and commercial applications of fungal chitosan: A review. J. Bioresour. Bioprod..

[91] Antunes F., Marcal S., Taofiq O., Morais A., Freitas A.C., Ferreira I., Pintado M. (2020). Valorization of Mushroom By-Products as a Source of Value-Added Compounds and Potential Applications. Molecules.

[92] Soultos N., Tzikas Z., Abrahim A., Georgantelis D., Ambrosiadis I. (2008). Chitosan effects on quality properties of Greek style fresh pork sausages. Meat Sci..

[93] Moon H., Kim N.H., Kim S.H., Kim Y., Ryu J.H., Rhee M.S. (2017). Teriyaki sauce with carvacrol or thymol effectively controls *Escherichia coli* O157:H7, *Listeria monocytogenes*, *Salmonella* Typhimurium, and indigenous flora in marinated beef and marinade. Meat Sci..

[94] Knez Hrnčič M., Španinger E., Košir I.J., Knez Ž., Bren U. (2019). Hop Compounds: Extraction Techniques, Chemical Analyses, Antioxidative, Antimicrobial, and Anticarcinogenic Effects. Nutrients.

[95] Mbandi E., Shelef L.A. (2001). Enhanced inhibition of *Listeria monocytogenes* and *Salmonella enteritidis* in meat by combinations of sodium lactate and diacetate. J. Food Prot..

[96] Lim K., Mustapha A. (2004). Effects of cetylpyridinium chloride, acidified sodium chlorite, and potassium sorbate on populations of *Escherichia coli* O157:H7, *Listeria monocytogenes*, and *Staphylococcus aureus* on fresh beef. J. Food Prot..

[97] Mancini R.A., Ramanathan R., Hunt M.C., Kropf D.H., Mafi G.G. (2022). Interrelationships Between Visual and Instrumental Measures of Ground Beef Color. Meat Muscle Biol..

[98] Kerth C.R., Miller R.K. (2015). Beef flavor: A review from chemistry to consumer. J. Sci. Food Agric..

[99] Chen T.C., Waimaleongora-Ek C. (1981). Effect of pH on TBA Values of Ground Raw Poultry Meat. J. Food Sci..

[100] Dominguez R., Pateiro M., Gagaoua M., Barba F.J., Zhang W., Lorenzo J.M. (2019). A Comprehensive Review on Lipid Oxidation in Meat and Meat Products. Antioxidants.

[101] Pohlman F.W., Stivarius M.R., McElyea K.S., Johnson Z.B., Johnson M.G. (2002). The effects of ozone, chlorine dioxide, cetylpyridinium chloride and trisodium phosphate as multiple antimicrobial interventions on microbiological, instrumental color, and sensory color and odor characteristics of ground beef. Meat Sci..

[102] EFSA BIOHAZ Panel (EFSA Panel on Biological Hazards) (2016). Scientific opinion on the growth of spoilage bacteria during storage and transport of meat. EFSA J..

[103] Suman S.P., Mancini R.A., Joseph P., Ramanathan R., Konda M.K., Dady G., Yin S. (2010). Packaging-specific influence of chitosan on color stability and lipid oxidation in refrigerated ground beef. Meat Sci..

[104] Amoli P.I., Hadidi M., Hasiri Z., Rouhafza A., Jelyani A.Z., Hadian Z., Khaneghah A.M., Lorenzo J.M. (2021). Incorporation of Low Molecular Weight Chitosan in a Low-Fat Beef Burger: Assessment of Technological Quality and Oxidative Stability. Foods.

[105] Hautrive T.P., Piccolo J., Rodrigues A.S., Campagnol P.C.B., Kubota E.H. (2019). Effect of fat replacement by chitosan and golden flaxseed flour (wholemeal and defatted) on the quality of hamburgers. LWT Food Sci. Technol..

[106] Sagheer F.A.A., Al-Sughayer M.A., Muslim S., Elsabee M.Z. (2009). Extraction and characterization of chitin and chitosan from marine sources in Arabian Gulf. Carbohydr. Polym..

[107] Qin Y.Y., Yang J.Y., Lu H.B., Wang S.S., Yang J., Yang X.C., Chai M., Li L., Cao J.X. (2013). Effect of chitosan film incorporated with tea polyphenol on quality and shelf life of pork meat patties. Int. J. Biol. Macromol..

[108] Khan I., Tango C.N., Oh D.H. (2017). Development and evaluation of chitosan and its derivative for the shelf life extension of beef meat under refrigeration storage. Int. J. Food Sci. Technol..

[109] Hong V., Wrolstad R.E. (1986). Cranberry juice composition. J. Assoc. Off. Anal. Chem..

[110] Wu V.C., Qiu X., de los Reyes B.G., Lin C.S., Pan Y. (2009). Application of cranberry concentrate (*Vaccinium macrocarpon*) to control *Escherichia coli* O157:H7 in ground beef and its antimicrobial mechanism related to the downregulated *slp*, *hdeA* and *cfa*. Food Microbiol..

[111] Hulankova R., Borilova G., Steinhauserova I. (2013). Combined antimicrobial effect of oregano essential oil and caprylic acid in minced beef. Meat Sci..

[112] Sayas-Barbera E., Quesada J., Sanchez-Zapata E., Viuda-Martos M., Fernandez-Lopez F., Perez-Alvarez J.A., Sendra E. (2011). Effect of the molecular weight and concentration of chitosan in pork model burgers. Meat Sci..

[113] Knorr D. (1982). Functional Properties of Chitin and Chitosan. J. Food Sci..

[114] Dong J., Kou X., Liu L., Hou L., Li R., Wang S. (2021). Effect of water, fat, and salt contents on heating uniformity and color of ground beef subjected to radio frequency thawing process. Innov. Food Sci. Emerg. Technol..

[115] Darmadji P., Izumimoto M. (1994). Effect of chitosan in meat preservation. Meat Sci..

[116] Huang X., Ahn D.U. (2019). Lipid oxidation and its implications to meat quality and human health. Food Sci. Biotechnol..

[117] Dunshea F.R., D’Souza D.N., Pethick D.W., Harper G.S., Warner R.D. (2005). Effects of dietary factors and other metabolic modifiers on quality and nutritional value of meat. Meat Sci..

[118] McKenna D.R., Mies P.D., Baird B.E., Pfeiffer K.D., Ellebracht J.W., Savell J.W. (2005). Biochemical and physical factors affecting discoloration characteristics of 19 bovine muscles. Meat Sci..

[119] Campo M.M., Nute G.R., Hughes S.I., Enser M., Wood J.D., Richardson R.I. (2006). Flavour perception of oxidation in beef. Meat Sci..

[120] Muthu M., Gopal J., Chun S., Devadoss A.J.P., Hasan N., Sivanesan I. (2021). Crustacean Waste-Derived Chitosan: Antioxidant Properties and Future Perspective. Antioxidants.

[121] Alimi B.A., Pathania S., Wilson J., Duffy B., Frias J.M.C. (2023). Extraction, quantification, characterization, and application in food packaging of chitin and chitosan from mushrooms: A review. Int. J. Biol. Macromol..

[122] Tikhonov V.E., Stepnova E.A., Babak V.G., Yamskov I.A., Palma-Guerrero J., Jansson H.-B., Lopez-Llorca L.V., Salinas J., Gerasimenko D.V., Avdienko I.D. (2006). Bactericidal and antifungal activities of a low molecular weight chitosan and its N-/2(3)-(dodec-2-enyl)succinoyl/-derivatives. Carbohydr. Polym..

[123] Duran A., Kahve H.I. (2020). The effect of chitosan coating and vacuum packaging on the microbiological and chemical properties of beef. Meat Sci..

[124] Jeremiah L.E. (2001). Packaging alternatives to deliver fresh meats using short- or long-term distribution. Food Res. Int..

[125] Lee C.H., Reed J.D., Richards M.P. (2006). Ability of Various Polyphenolic Classes from Cranberry to Inhibit Lipid Oxidation in Mechanically Separated Turkey and Cooked Ground Pork. J. Muscle Foods.

[126] Aguiar Campolina G., das Gracas Cardoso M., Rodrigues-Silva-Caetano A., Lee Nelson D., Mendes Ramos E. (2023). Essential Oil and Plant Extracts as Preservatives and Natural Antioxidants Applied to Meat and Meat Products: A Review. Food Technol. Biotechnol..

[127] Belles M., Alonso V., Roncales P., Beltran J.A. (2019). Sulfite-free lamb burger meat: Antimicrobial and antioxidant properties of green tea and carvacrol. J. Sci. Food Agric..

[128] Serio A., Chaves-Lopez C., Sacchetti G., Rossi C., Paparella A. (2018). Chitosan Coating Inhibits the Growth of *Listeria Monocytogenes* and Extends the Shelf Life of Vacuum-Packed Pork Loins at 4 °C. Foods.

