# Supplementary material for: Potential Use of Selected Natural Anti-Microbials to Control Listeria monocytogenes in Vacuum Packed Beef Burgers and Their Impact on Quality Attributes
Source: Microorganisms. 2025 Apr 16;13(4):910. doi: 10.3390/microorganisms13040910 (PMC12029336; doi:10.3390/microorganisms13040910)
Supplement: Supplementary file 1 [file microorganisms-13-00910-s001.zip › microorganisms-3479765-supplementary.pdf]

Table S1. Minimum Inhibitory and Bactericidal Concentrations (MIC and MBC) of a range of natural anti-microbial agents against *L. monocytogenes* under different conditions of growth.

Anti-microbial agent	Concentration range	T (˚C)	pH	Strain	MIC (%)	MBC (%)
Chitosan (Shrimp)	0.002 - 1% w/v	37	7.3	A, B, C, D	0.031	0.063
				E	0.031	0.125
		37	4.5	A, B, E	ND	0.125
				C	ND	0.25
				D	ND	0.063
		4	7.3	A, B, E	ND	0.25
				C, D	ND	0.5
		4	4.5	A, B, D, E	ND	0.25
				C	ND	0.5
Chitosan (Mushroom)	0.002 – 1% w/v	37	7.3	A, B	0.031	0.063
				C, E	0.031	0.031
				D	0.016	0.031
		37	4.5	A, C	ND	0.125
				B, D	ND	0.031
				E	ND	0.25
		4	7.3	A, C	ND	1
				B, D, E	ND	0.5
		4	4.5	A, D	ND	0.125
				B	ND	0.063
				C, E	ND	0.25
Carvacrol	0.000195 - 0.2% v/v	37	7.3	A	0.1	0.1
				B, C, D, E	0.05	0.1
		37	4.5	A, B, C, D, E	ND	0.05
		4	7.3	A, B, C, D, E	ND	0.1

		4	4.5	A, B, C<br>D, E	ND<br>ND	0.1<br>0.05
Thyme EO	0.002 – 1% v/v	37	7.3	A, B, C, D, E	0.125	0.125
		37	4.5	A, B, C, D, E	ND	0.063
		4	7.3	A, B, C, D, E	ND	0.125
		4	4.5	A, C, D, E<br>B	ND<br>ND	0.125<br>0.063
Rosemary EO	0.016 – 8% v/v	37	7.3	A, C, D<br>B<br>E	1<br>1<br>1	4<br>2<br>8
		37	4.5	A, E<br>B, C<br>D	ND<br>ND<br>ND	2<br>1<br>0.5
		4	7.3	A, B, C, D, E	ND	> 8
		4	4.5	A, C, D, E<br>B	ND<br>ND	> 8<br>4
Clove EO	0.004 – 2% v/v	37	7.3	A, B, C, D, E	0.25	0.25
		37	4.5	A, B, C, D, E	ND	0.063
		4	7.3	A<br>B, C, D, E	ND<br>ND	0.5<br>0.25
		4	4.5	A, B, C, D, E	ND	0.25
Cinnamon EO	0.004 – 2% v/v	37	7.3	A, B<br>C, D<br>E	0.063<br>0.063<br>0.125	0.125<br>0.25<br>0.125
		37	4.5	A, C, E<br>B<br>D	ND<br>ND<br>ND	0.125<br>0.031<br>0.063
		4	7.3	A, D, E<br>B	ND<br>ND	2<br>0.5

				C	ND	1
		4	4.5	A, B, C, D, E	ND	> 2
Hop extract	0.000098 - 0.4% v/v	37	7.3	A, E	0.002	0.4
				B	0.003	0.4
				C	0.003	0.2
				D	0.001	> 0.4
		37	4.5	A	ND	0.001
				B, D, E	ND	0.0004*
				C	ND	0.0002*
		4	7.3	A, B, C, D, E	ND	> 0.4
		4	4.5	A, B	ND	0.05
				C, E	ND	0.1
				D	ND	> 0.4
Cranberry extract	0.04 – 20% w/v	37	7.3	A, C, D, E	0.625	5
				B	0.625	1.25
		37	4.5	A, B, C, D, E	ND	1.25
		4	7.3	A, C, D, E	ND	20
				B	ND	10
		4	4.5	A, C, D, E	ND	10
				B	ND	5
Cranberry pomace	0.02 - 10% w/v	37	7.3	A, B, C, D, E	1.25	> 10
		37	4.5	A, B, C, D, E	ND	10
		4	7.3	A, B, C, D, E	ND	> 10
		4	4.5	A, B, C, D, E	ND	> 10
Propolis extract	0.02 – 1% w/v	37	7.3	A	0.125	0.5
				B, C, E	0.125	0.25
				D	0.125	0.125
		37	4.5	A, B, C, D	ND	0.031
				E	ND	0.063

	4	7.3	A, B, C, D, E	ND	> 1
	4	4.5	A, B, C, D, E	ND	> 1

ND = Not Determined as no growth was observed; A = *L. monocytogenes* NCTC 11994; B = *L. monocytogenes* Scott A; C = *L. monocytogenes* 2081; D = *L. monocytogenes* EGD-e; E = *L. monocytogenes* 3,104; *These MBC were rounded to 4 decimal places.

Table S2. Effect sizes (η^2) for the impact of natural anti-microbial agents on the visual colour, off-odour, overall odour, and overall quality of vacuum packed beef burgers during chilled storage (at 0 min after opening the packaging).

	**Visual colour**			**Off-odour**			**Overall odour**			**Overall quality**		
**Variable**	***P* value** [1]	**η^2**	**η^2 95% Confidence Interval**	***P* value**	**η^2**	**η^2 95% Confidence Interval**	***P* value**	**η^2**	**η^2 95% Confidence Interval**	***P* value**	**η^2**	**η^2 95% Confidence Interval**
Anti-microbial agent	<0.0001	0.62	[0.56, 0.67]	0.2903	0.02	[0, 0.04]	<0.0001	0.09	[0.03, 0.14]	<0.0001	0.35	[0.27, 0.42]
Storage day	<0.0001	0.06	[0.02, 0.12]	0.1842	0.01	[0, 0.04]	0.7469	0.00	[0, 0.02]	0.1977	0.01	[0, 0.04]
Anti-microbial agent: Storage day	0.4672	0.02	[0, 0.04]	0.0251	0.05	[0, 0.08]	0.6670	0.02	[0, 0.03]	0.7933	0.01	[0, 0.02]

[1] Statistical significance was set at $P \leq 0.05$.

Table S3. Effect sizes (η^2) for the impact of natural anti-microbial agents on the visual colour, off-odour, overall odour, and overall quality of vacuum packed beef burgers during chilled storage (at 30 min after opening the packaging).

	**Visual colour**			**Off-odour**			**Overall odour**			**Overall quality**		
**Variable**	***P* value** [1]	**η^2**	**η^2 95% Confidence Interval**	***P* value**	**η^2**	**η^2 95% Confidence Interval**	***P* value**	**η^2**	**η^2 95% Confidence Interval**	***P* value**	**η^2**	**η^2 95% Confidence Interval**
Anti-microbial agent	<0.0001	0.59	[0.53, 0.65]	0.0296	0.03	[0, 0.07]	<0.0001	0.08	[0.03, 0.13]	<0.0001	0.38	[0.3, 0.45]
Storage day	0.0013	0.04	[0.01, 0.09]	0.8815	0.00	[0, 0.01]	0.7521	0.00	[0, 0.02]	0.0545	0.02	[0, 0.05]
Anti-microbial agent: Storage day	0.3360	0.03	[0, 0.05]	0.0222	0.05	[0, 0.09]	0.7189	0.02	[0, 0.03]	0.1323	0.04	[0, 0.06]

[1] Statistical significance was set at $P \leq 0.05$.
